# LUBAC promotes angiogenesis and lung tumorigenesis by ubiquitinating and antagonizing autophagic degradation of HIF1α

**DOI:** 10.1038/s41389-024-00508-3

**Published:** 2024-01-25

**Authors:** Ying Jin, Yazhi Peng, Jie Xu, Ye Yuan, Nan Yang, Zemei Zhang, Lei Xu, Lin Li, Yulian Xiong, Dejiao Sun, Yamu Pan, Ruiqing Wu, Jian Fu

**Affiliations:** 1 The Laboratory of Inflammation and Vascular Biology, Institute of Clinical Medicine and Department of Cardiology, Renmin Hospital, Hubei University of Medicine, Hubei, China; 2 Aab Cardiovascular Research Institute and Department of Medicine, University of Rochester School of Medicine and Dentistry, Rochester, NY USA; 3 College of Life Science and Technology, Huazhong University of Science and Technology, Wuhan, China; 4 Graduate School, Jinzhou Medical University, Liaoning, China

**Keywords:** Ubiquitylation, Ubiquitylation, Tumour angiogenesis

## Abstract

Hypoxia-inducible factor 1 (HIF1) is critically important for driving angiogenesis and tumorigenesis. Linear ubiquitin chain assembly complex (LUBAC), the only known ubiquitin ligase capable of catalyzing protein linear ubiquitination to date, is implicated in cell signaling and associated with cancers. However, the role and mechanism of LUBAC in regulating the expression and function of HIF1α, the labile subunit of HIF1, remain to be elucidated. Herein we showed that LUBAC increases HIF1α protein expression in cultured cells and tissues of human lung cancer and enhances HIF1α DNA-binding and transcriptional activities, which are dependent upon LUBAC enzymatic activity. Mechanistically, LUBAC increases HIF1α stability through antagonizing HIF1α decay by the chaperone-mediated autophagy (CMA)-lysosome pathway, thereby potentiating HIF1α activity. We further demonstrated that HIF1α selectively interacts with HOIP (the catalytic subunit of LUBAC) primarily in the cytoplasm. LUBAC catalyzes linear ubiquitination of HIF1α at lysine 362. Linear ubiquitination shields HIF1α from interacting with heat-shock cognate protein of 70 kDa and lysosome-associated membrane protein type 2 A, two components of CMA. Consequently, linear ubiquitination confers protection against CMA-mediated destruction of HIF1α, increasing HIF1α stability and activity. We found that prolyl hydroxylation is not a perquisite for LUBAC’s effects on HIF1α. Functionally, LUBAC facilitates proliferation, clonogenic formation, invasion and migration of lung cancer cells. LUBAC also boosts angiogenesis and exacerbates lung cancer growth in mice, which are greatly compromised by inhibition of HIF1α. This work provides novel mechanistic insights into the role of LUBAC in regulating HIF1α homeostasis, tumor angiogenesis and tumorigenesis of lung cancer, making LUBAC an attractive therapeutic target for cancers.

## Introduction

Ubiquitination is a versatile post-translational modification of proteins that is orchestrated by the concerted action of E1 ubiquitin (Ub) activating enzyme, E2 Ub conjugating enzyme, and E3 Ub ligase [1]. The linear Ub chain assembly complex (LUBAC) is a Ub ligase capable of catalyzing specifically protein linear ubiquitination [2]. LUBAC is best known for its ability to regulate immunity, inflammation and cell death through orchestrating NF-κB signaling [2]. We are far from a comprehensive understanding of LUBAC in cellular signaling to date [2]. LUBAC has been linked to diverse diseases including cancers [3–5]. Nonetheless, the mechanisms whereby LUBAC dictates tumorigenesis remain elusive.

LUBAC is the only known E3 ligase identified to date that can assemble linear Ub chains [6–9]. LUBAC comprises three subunits: a large isoform of heme-oxidized iron regulatory protein 2 (IRP2) Ub ligase 1 (HOIL-1L), HOIL-1L interacting protein (HOIP), and SHANK-associated RH domain-interacting protein (Sharpin) [6–9]. The catalytic activity of LUBAC is rendered by HOIP [7, 8]. HOIP alone is not sufficient to generate linear Ub chains; it requires HOIL-1L and/or Sharpin to assemble functional LUBAC [7, 8, 10]. OTU deubiquitinase with linear linkage specificity (Otulin) is the only mammalian deubiquitinating enzyme pruning linear Ub chains from proteins [11, 12].

Hypoxia-inducible factor 1 (HIF1) consists of a labile HIF1α and a constitutively expressed HIF1β subunits [13, 14]. The Ub-proteasome system (UPS) is the best-characterized mechanism regulating the stability of HIF1α [15, 16]. In well-oxygenated cells, HIF1α is hydroxylated at prolines by the EGLN family of prolyl hydroxylases [17, 18]. Hydroxylated proline residues provide the docking sites for the von Hippel-Lindau protein (pVHL), leading to HIF1α lysine (K)48-ubiquitination and proteasomal degradation [17, 18]. Blockade of HIF1α hydroxylation results in its accumulation [18]. HIF1α then translocates into the nuclei, where it dimerizes with HIF1β and binds to the hypoxia response elements (HREs) in target genes to fuel transcription [19]. Thus, prolyl hydroxylation is fundamentally important for HIF1α ubiquitination and decay. Besides pVHL, growing lists of E3 Ub ligases target HIF1α for ubiquitination [15, 16]. The role for LUBAC in inducing HIF1α linear ubiquitination has not been reported until now. Besides the UPS, recent findings demonstrated that chaperone-mediated autophagy (CMA) contributes significantly to HIF1α proteolysis [20, 21]. In the CMA pathway, heat-shock cognate protein of 70 kDa (HSC70) recognizes a substrate and then delivers the cargo to lysosome-associated membrane protein type 2 A (LAMP2A) for destruction [22–24]. However, the regulatory mechanism behind CMA-mediated HIF1α proteolysis is poorly defined.

Lung carcinoma is the leading cause of cancer-related deaths worldwide [25]. LUBAC is upregulated in cancers including lung cancer [26]. However, the role and mechanism for LUBAC in lung cancer are elusive. Angiogenesis, the sprouting of new capillaries from pre-existing vessels, is fundamentally important for tumor growth and development [13]. Given the crucial role for angiogenesis in the growth and progression of lung cancer, anti-angiogenic drugs have been approved by the Food and Drug Administration for treatment of patients with non-small cell lung cancer [27]. However, the therapeutic benefits are varied and this strategy is challenged by significant side effects [28]. Thus, more studies are warranted to identify new molecules and mechanisms for the control of tumor angiogenesis in order to develop novel strategy for the treatment of lung cancer. Intriguingly, genetic evidence showed that LUBAC and linear ubiquitination dictate embryonic vascularization [12, 29, 30]. Further mechanistic study indicated that LUBAC regulates embryonic vascularization through inducing the linear ubiquitination of activin receptor-like kinase [30]. To the best of our knowledge, it has not been reported whether LUBAC regulates tumor angiogenesis to date. Hypoxia-inducible factor 1 (HIF1) is the master regulator of angiogenesis by activating the expression of proangiogenic genes, notably vascular endothelial growth factor (VEGF) [13, 14]. It remains to be determined whether LUBAC dictates tumor angiogenesis through targeting HIF1 signaling. Given the critical role for angiogenesis in cancers, this study aimed to decipher the role and mechanism for LUBAC in HIF1α linear ubiquitination, stability, angiogenesis and tumorigenesis.

## Materials and methods

### DNA Constructs

To make vectors expressing full-length or different fragments of HIF1α with Flag, myc, GFP, or glutathione S-transferase (GST) tags, PCR amplification was performed using pfu polymerase (Yeasen) and the amplicons were inserted into p3xflag-CMV-13, pCDNA3-myc, pEGFP-C1, or pEBG. The constructs harboring EGFP-tagged HOIP and mCherry-tagged HIF1α were generated by cloning the DNA fragments encoding HOIP-EGFP and HIF1α-mCherry into p3xflag-CMV-13. Lamin B1 cDNA was subcloned into pTagBFP-C1 to construct plasmid expressing Lamin B1-BFP. The lentivirus expressing HOIP was engineered by cloning HOIP cDNA into pLvx-t2a-mCherry. The constructs harboring HA-tagged Otulin, CYLD or A20 were obtained by subcloning the PCR products into pCDNA3.1. The plasmid expressing GST-specific tandem Ub-binding entity (TUBE) was constructed by subcloning the cDNA encoding UBAN domain of IκB kinase (IKK) γ into pGEX-4T-1. Site-directed mutagenesis of HIF1α mutants was conducted to generate different point mutants using site-directed mutagenesis kit (Yeasen) as described previously [31, 32]. Other plasmids were described in the previous publications [6, 31–39]. All constructs were verified by DNA sequencing.

### RNA interference

LAMP2A small interfering RNAs (*siRNAs*) (5’-CGCUAUGAAACUACAAAUATT-3’; 5’-GCUCUACUUAGACUCAAUATT-3’), *HIF1α siRNAs* (5’-GCUAUUCACCAAAGUUGAATT-3’; 5’-CAUGAAAGCACAGAUGAAUTT-3’), *HOIP siRNAs* (5’-CGUGGUGUAAAGUUUAAUATT-3’; 5’-GGCGUGGUGUCAAGUUUAATT-3’), *HOIL-1L siRNAs* (5’CACACCUUCUGCAGGGAGUTT-3’; 5’-ACUCCCUGCAGAAGGUGUGTT-3’), *Sharpin siRNAs* (5’-CCUGGAAACUUGACGGAGATT-3’; 5’-CUGCUUUCCUCUACUUGCUTT-3’) and *Otulin siRNAs* (5’-GACUGAAAUUUGAUGGGAATT-3’; 5’-CAAAUGAGGCGGAGGAAUATT-3’), and control siRNAs were purchased from RIBOBIO (Guangzhou, China). *HOIP siRNA* was also purchased from Santa Cruz Biotechnology (sc-92101). The siRNAs were transfected into cells using Lipofectamine 2000 reagent (Invitrogen) as described previously [32, 38].

### Antibodies

anti-FLAG M2 (Sigma, F1804), anti-HA.11 (BioLegend, 901501), anti-myc (Santa Cruz Biotechnology, sc-40), anti-GST (Yeasen, 30902ES60), anti-α-tubulin (Yeasen, 30304ES60), anti-β-actin (Yeasen, 30101ES60), anti-GAPDH (Immunoway, YM3029), anti-CD31 (Abcam, ab28364), anti-VEGF (Novus Biologicals, NB100-664), anti-PCNA (Servicebrio, GB11010), anti-ubiquitin (Santa Cruz Biotechnology, sc-8017), anti-GFP (Yeasen, 31002ES60), anti-LUB9 (Lifesensors, AB130), anti-HIF1α (Novus, NB100-479; Abcam, ab228649), anti-HIF1β (Cell Signaling technology, #5537), anti-Otulin (Abcam, ab211328), anti-HOIP (Abcam, ab46322; R&D systems, MAB8039), anti-HOIL-1L (Millipore, MABC576), anti-Sharpin (Proteintech, 14626-1-AP), anti-LAMP2 (Santa Cruz Biotechnology, sc-18822), anti-Lamin B1 (Zenbio, R24825), normal mouse immunoglobulin (IgG) 1 (Santa Cruz Biotechnology, sc-3877), anti-mouse IgG(H + L) (Jackson ImmunoResearch, 151383), and anti-rabbit IgG(H + L) (Jackson ImmunoResearch, 145472).

### Cell culture and transfection

Human embryonic kidney (HEK) 293, HEK293T, A549, NCI-H460, and J774A cells were obtained from ATCC (Manassas, VA). HEK293, HEK293T, A549 and J774A cells were maintained in Dulbecco’s Modified Eagle’s Medium (DMEM) supplemented with 10% heat-inactivated fetal bovine serum (FBS). NCI-H460 cells were maintained in RPMI 1640 medium supplemented with 10% FBS. Hypoxic culture of cells was conducted as described previously [31, 37]. All cell lines were verified to be mycoplasma-free. Cells were transfected as described previously [40].

### Preparation of bone marrow-derived macrophages (BMDMs)

Mouse BMDMs were prepared from *hoip*^*fl/fl*^ mice and differentiated into macrophages as described previously [40, 41]. *Hoip*^*fl/fl*^ mouse strain [42] was kindly provided by Prof. X. Lin.

### Luciferase reporter assay

Luciferase expression in cells transfected was measured using a Dual-Luciferase reporter assay system (Yeasen) following the manufacturer’s instructions. The expression of firefly luciferase driven by the HIF1-HRE was used as a reporter. *pRL-tk* (renilla luciferase) was cotransfected to normalize for the transfection efficiency. Luciferase activity was expressed as a ratio of firefly luciferase activity to renilla luciferase activity. Normalized values are reported as the means ± SEM (standard error of the mean) of the results of triplicate transfection. Student’s *t-*test for paired samples was used to determine statistical significance.

### Establishment of stable A549 overexpressing HOIP cell line

HEK293T cells were co-transfected using Lipofectamine 2000 with lentiviral vector (*plvx-Flag-HOIP*) and packaging plasmids (*plp1*, *plp2* and *vsvg*). After 24 h and 48 h posttransfection, the supernatants containing lentiviral particles were harvested and filtered through 0.45-μm filters. A549 cells were infected by the letivirus particles and then selected with puromycin (Invivogen). The single cell-derived colonies were isolated, expanded and analyzed by IB.

### Establishment of A549 HOIP knockout cell lines

A549 HOIP knockout (A549^HOIPKO^) cell lines were established using CRISPR/Cas9 technology. Sequences of three guide RNAs used were: *sgRNA1*-F (CACCGTGACTCCTGCCTCAGGATGC); *sgRNA2*-F (CACCGTGACTCCTGCCTCAGGATGC); *sgRNA3*-F (CACCGTTGACACCACGCCAGTACCG). gRNA oligonucleotides were cloned into lentiCRISPRv2. A549 cells were infected by the letivirus particles from HEK293T cells transfected with the constructs containing *sgRNAs*, *psPAX2* and *pMD2.G*. The cells were selected using puromycin. The single colonies were isolated, expanded and analyzed by IB.

### Real-time quantitative PCR (qPCR)

Total RNA was extracted from the cells using Trizol reagent (Yeasen) per the manufacturer’s instructions. Reverse transcription of template RNA into cDNA was performed using a RevertAid First Strand cDNA Synthesis Kit (Thermo Scientific). For quantitative PCR (qPCR) analysis of gene expression, amplification was conducted using a FastStart Universal SYBR Green Master (Roch) and run on a Real‐time PCR System (ABI‐7000). All samples were run in triplicate. The Ct values for target genes and the reference gene were recorded. The expression level of GAPDH was used for normalization. Primer sequences are available upon request.

### Immunoblotting (IB)

IB was carried out as previously described [31, 36–39]. Cellular proteins were quantified, resolved on SDS-PAGE and electroblotted onto a polyvinylidene difluoride membrane. Following blocking, the membrane was incubated with an appropriate primary antibody and then incubated with a corresponding anti-mouse IgG or anti-rabbit IgG conjugated to horse radish peroxidase. The blots were developed by ECL (Yeasen) or ECL Plus (Yeasen) method.

### Immunoprecipitation (IP)

IP was conducted as previously described [31]. The precleared lysates were incubated with the corresponding antibody (about 1–1.5 μg each) in the presence of 20 μl of Protein A/G Agarose (Thermo Scientific) overnight with constant agitation. After extensive washing, the immunoprecipitates were subjected to IB. For in vivo ubiquitination assays [38], denatured IP was conducted. In brief, cells were first solubilized in lysis buffer supplemented with 1% SDS and boiled for 10 min. The denatured lysates were diluted with lysis buffer (without SDS) followed by IP/IB as described above.

### GST pulldown assay

GST pulldown assay was performed as previously described [31, 32]. Cells were extracted in NETN buffer [31, 32]. About 500-700 μg of cell lysates were mixed with 20 μl of glutathione agarose resin in NETN buffer with protease inhibitors. After extensive washing, the complexes were eluted with SDS sample buffer and detected by IB.

### Expression and purification of GST-TUBE

Cultures of *Escherichia coli* BL21 (DE3) pLysS transformed with *pGEX4T-1* or *pGEX4T-1* containing GST-TUBE were grown at room temperature (RT) with shaking to an OD600 of 0.6. Isopropyl-D-thiogalactopyranoside (Beyotime) was then added to reach a final concentration of 0.5 mM. After an additional 12 h of growth, cells were harvested in GST binding buffer [38]. After sonication, Triton X-100 was added to reach a final concentration of 1%. The GST fusion proteins were adsorbed to glutathione agarose resin.

### GST-TUBE assay

Linear Ub conjugates were purified from cell lysates using purified GST-TUBE [43]. Cells were harvested in NETN buffer supplemented with 5 mM N-ethylmaleimide and protease inhibitors. GST-TUBE (100 μg/ml) pre-bound to glutathione agarose beads were incubated with cell lysates. After extensive washing, the bound material was eluted with sample loading buffer.

### Purification of HA-HIF1α and its mutants

HA-HIF1α and HA-HIF1α^K362R^ were transiently expressed in HEK293T cells. HEK293 cells stably expressing HA-HIF1α^PA^ and HA-HIF1α^PAK362R^ were generated. Cells were extracted in buffer A (20 mM Tris [pH 7.5], 100 mM NaCl, 1 mM EDTA, and protease inhibitors). Cleared lysates were immunoprecipitated with anti-HA. The immunoprecipitates were washed sequentially with buffer B (20 mM Tris [pH7.5], 420 mM NaCl, 1.5 mM MgCl_2_, 0.2 mM EDTA, 25% glycerol, and protease inhibitors), buffer C (20 mM Tris [pH 7.5], 300 mM NaCl, 0.2 mM EDTA, 0.1% Igepal CA630, 20% glycerol, and protease inhibitors), and buffer D (50 mM Tris [pH7.5], 150 mM NaCl, and protease inhibitors). Samples were eluted with HA peptide (MCE) and the eluates concentrated by Amicon Ultra Centrifugal Filters.

### in vitro linear ubiquitination assay

The purified HA-HIF1α, HA-HIF1α^K362R^, HA-HIF1α^PA^ or HA-HIF1α^PAK362R^ (1 μg each) protein was incubated at 37 °C for 2 h with 30 μl of a Ub conjugation reaction buffer supplemented with 500 ng of Ub, 200 ng of E1, 500 ng of E2 and 1 μg of E3. The HOIP RING-in-between-RING (RBR) and linear Ub chain determining region (LDD) region of HOIP (HOIP-RBR-LDD) was used as the E3 ligase [44]. All reagents were purchased from BostonBiochem. The reaction was stopped by adding 2 x loading buffer, followed by boiling. The level of linear ubiquitination was monitored by IB with anti-LUB9.

### Chromatin immunoprecipitation (ChIP) assay

ChIP assay was conducted using the ChIP Kit (Beyotime) per the manufacturer’s protocol. Briefly, 1 × 10^7^ cells were cross-linked using 1% formaldehyde, quenched with glycine, lysed and sonicated to achieve a DNA shear length of 500 bp or so. Solubilized chromatin was diluted ten times in dilution buffer. Twenty microliters of the lysate were saved as the input control, and the remaining supernatant was incubated with anti-HIF1α or normal IgG as control overnight at 4 °C in the presence of protein A/G beads. After elution and reverse cross-linking, the eluted chromatin was treated with ribonuclease and proteinase K. Precipitated chromatin DNA was analyzed by qPCR. ChIP-qPCR primers were available upon request. All ChIP-qPCR data were normalized to those of IgG control, presented as fold enrichment and expressed as mean ± SEM. Student’s *t-*test for paired samples was used to determine statistical significance.

### Enzyme linked immunosorbent assay (ELISA)

The levels of VEGF in cell culture medium were determined using the ELISA kit (Neobioscience) according to the manufacturer’s instructions. Culture media was collected from cells, cleared by centrifugation at 12,000 *g* for 5–10 min and analyzed.

### Subcellular fractionation

Subcellular fractionation was performed using a Nuclear and Cytoplasmic Protein Extraction Kit (Beyotime) according to the manufacturer’s protocol with minor modifications. Cells were harvested, washed with PBS, and resuspended in Buffer A with protease inhibitors. After incubation for 15 min on ice, Buffer B was added and incubated on ice for an additional 1 min. The nuclei were separated by centrifugation at 14,000 *g* for 5 min at 4 °C. The supernatant was collected as cytosolic fraction. The pellet, containing the nuclei, was washed with PBS twice and then resuspended in radioimmune precipitation assay buffer (Millipore) containing 1 mM phenylmethylsulfonyl fluoride and protease inhibitor mixture for 10 min on ice. After centrifugation, the supernatant was collected as nuclear fraction. The cytosolic and nuclear fractions were analyzed by IB.

### Immunofluorescence staining

Immunostaining was conducted as previously described [31, 37]. Cells were fixed in 4% paraformaldehyde for 10 min at RT, permeabilized in PBST (PBS containing 0.1% Triton X-100) for 5–10 min at RT, and blocked in PBS with 1% bovine serum albumin. Cells were incubated with an anti-HOIP (R&D systems, MAB8039) at 4 °C overnight, followed by incubation with fluorescein isothiocyanate conjugated anti-mouse IgG for 45 min at RT. Following extensive washing with PBST, cells were probed with rabbit anti-HIF1α overnight at 4 °C, followed by incubation with Texas Red-conjugated anti-rabbit IgG for 45 min at RT. Cells were visualized by a fluorescent microscope.

### Confocal microscopy

For confocal microscopy, cells were co-transfected with plasmids expressing HOIP-EGFP, HIF1α-mCherry and Lamin B1-BFP. At 24 h of transfection, the cells were incubated in a cell culture environmental chamber (Tokai Hit, Japan) at 37 °C with 5% CO_2_. Images were acquired on an FV1000 confocal microscope (Olympus, Japan).

### Cycloheximide chase experiment

Cycloheximide (CHX) chase experiment was conducted as previously described [31]. Briefly, cells were treated with 100 μM of CHX (Yeasen) for the indicated time points, when the cells were harvested and analyzed by IB.

### Cell proliferation assay

Cell viability was determined by CCK8 assay using a commercial kit (Yeasen). Briefly, cells were seeded in 96-well plates (1×10^3^ cells/well) and treated as indicated in the figures. CCK8 was added into the wells for 3 h at indicated times. The absorbance in each well at wavelength of 450 nm (OD450) was measured with a Thermomax microplate reader.

### Transwell migration and invasion assays

For the migration assay, a total of 5 × 10^5^ cells in 100 μl of serum free medium per well were plated in the chamber inserts of 24-well Transwell plates (8-μm pore size, Corning), with medium containing 10% FBS at the bottom of the insert. For invasion assay, the inserts were matrigel-coated prior to seeding cells (5 × 10^5^ cells in 100 μl of serum free medium per well). Cells were incubated for 16 h (for migration assay) or 20 h (for invasion assay) at 37 °C. The cells were fixed with 4% paraformaldehyde and stained with 0.1% crystal violet. Cells on the upper surface of the insert were removed with a cotton swab. The migrated or invaded cells were counted under the microscope and statistically analyzed.

### Colony formation assay

Cancer cells were seeded in 6-well plates. Two weeks later, the cells were fixed with 4% paraformaldehyde and stained with 0.5% crystal violet solution. The cell colonies were counted and imaged.

### Endothelial cell (EC) tube formation assay

Human umbilical vein endothelial cells (HUVECs) were seeded into 24-well plate at 1×10^4^ per well pre-coated with matrigel (Corning). HUVECs were exposed to the conditioned medium of cells in Medium 200 (Gibco) supplemented with 1% FBS and low-serum growth supplement (LSGS)(Gibco). The plate was then incubated at 37 °C for 6 h and examined under an inverted microscope (Leica, Germany). The total branching points were calculated using NIH ImageJ software. Cells between passages 3 and 7 were used in this study.

### In vivo matrigel plug angiogenesis assay

All mouse procedures and experiments for this study were approved by the Institutional Animal Care and Use Committee of Renmin Hospital at the Hubei University of Medicine. Around 400 μl of matrigel in liquid form at 4 °C was mixed with equal volume of cells (1 × 10^7^ cells in PBS, and injected subcutaneously into of 6-week-old athymic nude mice). After 10 days, the mice were anesthetized by isoflurane and sacrificed. The plugs were removed and fixed with 10% formalin, embedded in paraffin, sectioned, and subjected to Masson’s trichrome staining or immune- staining with anti-CD31. Blood vessels were quantitated and statistically analyzed.

### Immunohistochemistry

Formalin-fixed paraffin-embedded tissues of human lung cancers were obtained from the Department of Pathology, Hubei University of Medicine Renmin Hospital, with an informed consent from the patients. The study was approved by the Institutional Health Research Ethics Board of Hubei University of Medicine Renmin Hospital. Mouse tissues were fixed in 60% methanol and 10% acetic acid in H_2_O (vol/vol) and embedded in paraffin. Tissue sections (5 μm) were subjected to immunohistochemical staining as previously described [32]. The positive staining of VEGF in tumor tissues were expressed as the mean of integrated optical density (IOD). IOD was acquired by calculation of the ratio of medium pixel intensity to the positive staining area using Image-Pro Plus software. At least 20 random 40x fields per mouse and 4–5 mice per group were analyzed. Data in graphs were presented as mean ± SEM. For quantification of PCNA and CD31 immunostaining, we counted the PCNA-positive cells or vessel numbers based on CD31 staining of at least ten random 40x fields per mouse and 4–5 mice per group were analyzed, which were conducted manually or using the Cell Counter function in NIH ImageJ. We averaged the results over the number of counted fields. Data in graphs were presented as the mean ± SEM. To minimize artificial effects, cells in necrotic areas, with poor morphology, and in the margins of sections were not taken into account in our study. The immunostaining results were assessed independently by 2 individuals in a blinded fashion.

### Tumor xenografts and tumor volume measurement

Six- to eight-week-old (18–20 g) female nude mice were purchased from Beijing Vital River Laboratory Animal Technology Co., Ltd (Beijing, China). Mice were randomly divided into two or three groups for each animal experiment. A total of 70 nude mice were used in our animal studies. The investigators were blinded to the group allocation during the experiment and/or when assessing the outcome. The animals that were injured, free of tumor, or died during the course of experiments were excluded from the analysis.

Xenograft transplantations were performed in a blind manner in nude mice according to the institutional guidelines and permissions for animal experiments, obtained from the regional authorities of the Hubei University of Medicine. 5 × 10^5^ cancer cells in 0.1 ml PBS were injected subcutaneously into the flanks of mice. Tumor dimensions were measured once when tumors were palpable. The tumor size was measured and the tumor volume was calculated using the formula: tumor volume = 0.5 × a^2 ^× b (where a is the short tumor diameter and b is the long tumor diameter). At the end of the experiment, mice were anesthetized by isoflurane. The mice were perfused via the left ventricle with 0.9% saline supplemented with heparin (50 U/mL), followed by another perfusion with 4% paraformaldehyde solution. The tumors were harvested and embedded in paraffin or optimal cutting temperature compound (OCT) (Sakura) and frozen in −80 °C for cryostats tissue sectioning. Tumors dissociated were subjected to immunohistochemical and IB analyses.

### Statistical analysis

Data analyses were conducted using GraphPad Prism 8.0 (GraphPad Software). Normal distribution was evaluated using the Shapiro-Wilk test. The variance was similar between the groups. Two-tailed Student’s *t*-test was applied to assess statistical differences between the 2 groups. Data are expressed as the mean ± SEM. The values of *p < 0.05 **p < 0.01 ***p < 0.001, and ****p < 0.0001 were considered statistically significant.

## Results

### LUBAC enhances the stability of the HIF1α protein in lung cancer

**Fig. 1:**
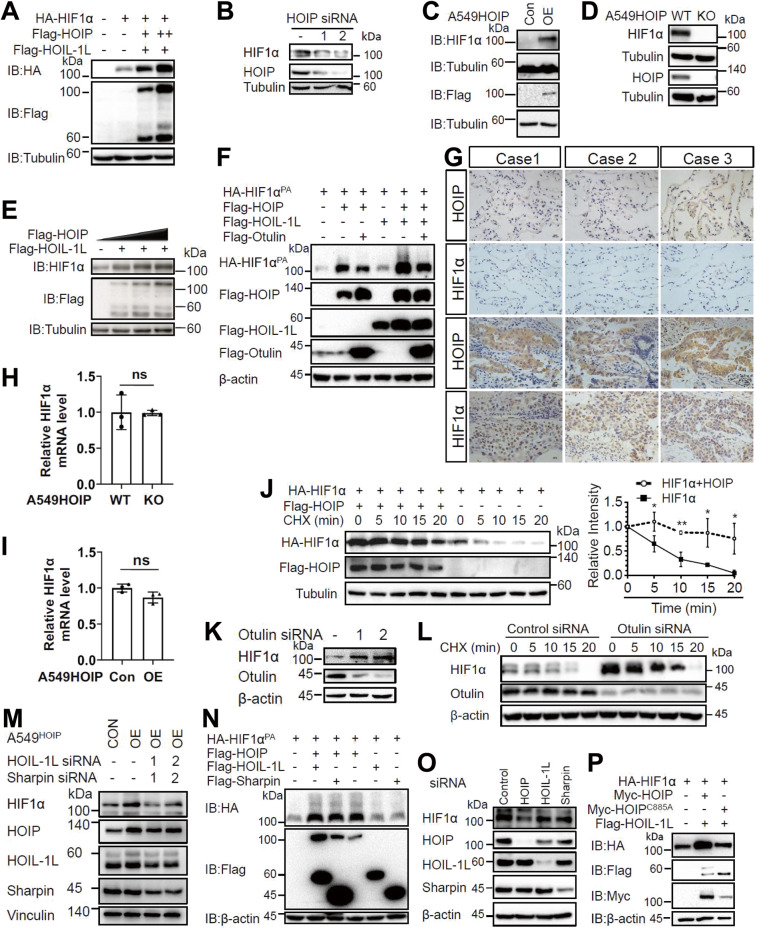
LUBAC-induced stabilization of the HIF1α protein requires its catalytic activity. **A** Immunoblotting (IB) analysis of the indicated proteins in HEK293T cells. **B** IB analysis of the indicated proteins in A549 cells transfected as indicated. **C**, **D** IB analysis of the indicated proteins in A549^HOIPCON^ and A549^HOIPOE^ (**C**) as well as A549^HOIPWT^ and A549^HOIPKO^ (**D**) cells. **E** IB analysis of the indicated proteins in HEK293T cells cultured under hypoxia for 8 h. **F** IB analysis of the indicated proteins in HEK293T cells transfected as indicated. **G** Immunohistochemistry analysis of human lung cancer and paracancerous normal tissues (*n* = 9). Scale bars: 150 μm. Original magnification: 400. **H**, **I** qPCR analysis of *HIF1α* mRNA relative to GAPDH in A549^HOIPWT^ and A549^HOIPKO^ (**H**) as well as A549^HOIPCON^ and A549^HOIPOE^ (**I**) cells (*n* = 3). **J** IB analysis of the indicated proteins in HEK293T cells transfected as indicated and then treated with cycloheximide (100 μM) for different time points (*n* = 3). **K** IB analysis of the indicated proteins in A549 cells transfected as indicated. **L** IB analysis of the indicated proteins in A549 cells transfected as indicated and then treated with cycloheximide for different time points (*n* = 3). **M** IB analysis of the indicated proteins in A549^HOIPCON^ and A549^HOIPOE^ cells transfected as indicated. **N** IB analysis of the indicated proteins in HEK293T cells transfected as indicated. **O** IB analysis of the indicated proteins in A549 cells transfected as indicated. **P** IB analysis of the indicated proteins in HEK293T cells transfected as indicated. Data are expressed as the mean ± SEM. *p < 0.05, **p < 0.01 by *p*aired 2-tailed Student’s *t*-test.

LUBAC is involved in NF-кB signaling, but its role in HIF signaling remains unknown. Interestingly, we found that overexpression of LUBAC significantly enhanced HIF1α expression (Fig. 1A and S1A). Notably, overexpression of HOIP alone dramatically increased HIF1α expression in HEK293T cells (Fig. S1B), which is ascribed to high expression of HOIL-1L and Sharpin in cells [8]. Knockdown of HOIP with two pairs of *HOIP* siRNA reduced HIF1α expression in A549 lung carcinoma cells (Fig. 1B) and HEK293T cells (data not shown). Furthermore, we generated A549 cell lines stably expressing HOIP (termed A549^HOIPOE^ cells) (Fig. 1C) and *HOIP* gRNAs (designated A549^HOIPKO^ cells) (Fig. 1D), respectively. Overexpression of HOIP significantly enhanced HIF1α expression in normoxia (Fig. 1M) and hypoxia (Fig. 1C), whereas knockout of HOIP decreased HIF1α levels (Fig. 1D) in lung cancer cells. Likewise, elimination of HOIP also decreased HIF1α expression in NCI-H460 lung cancer cells (Fig. S1C), and mouse macrophages (Fig. S1D). Thus, LUBAC positively regulates HIF1α expression in different types of cells examined. Transient overexpression of HOIP also markedly induced HIF1α expression under hypoxia (Fig. 1E). Furthermore, LUBAC enhanced the expression of HIF1α^PA^ (Fig. 1F), a prolyl hydroxylation-resistant mutant. In sum, prolyl hydroxylation is dispensable for the HIF1α-stabilizing effect of LUBAC. To further confirm the aforementioned findings, we performed immunostaining analysis of HOIP and HIF1α expression in human lung cancer tissues. We found that HOIP expression was elevated in lung cancers compared with the adjacent normal tissues, which was correlated with HIF1α expression (Fig. 1G).

qPCR showed that the expression of *HIF1α* mRNA was not overtly altered in A549^HOIPKO^ (Fig.1H) and A549^HOIPOE^ (Fig. 1I) cells. Thus, up-regulation of HIF1α expression by HOIP did not occur at the transcriptional level. In keeping with these observations, HOIP significantly increased HIF1α protein stability (Fig. 1J), as judged by cycloheximide (CHX) chase experiment. Together, LUBAC stabilizes HIF1α protein in lung cancer.

### Increase of HIF1α expression by LUBAC requires its catalytic activity

We next determined whether the catalytic activity of LUBAC is essential for its HIF1α-stabilizing effect. Otulin is a deubiquitinase selectively trimming the linear Ub chains [11, 12]. Thus, we tested the role of Otulin in regulating HIF1α-stabilizing effect of LUBAC. Deletion of Otulin with two pairs of siRNAs increased HIF1α expression in A549 (Fig. 1K) and NCI-H460 (Fig. S1E) lung cancer cells. Notably, Otulin silencing had no discernible effect on *HIF1α* mRNA abundance (Fig. S1F). Importantly, deletion of Otulin enhanced HIF1α protein stability in A549 cells (Fig. 1L). HOIP-induced HIF1α expression was efficiently counteracted by Otulin but not by another deubiquitinase CYLD (Fig. 1F and S1G).

Since HOIL-1L and/or Sharpin are essential for LUBAC catalytic activity [6–9], we next examined whether they contribute to HIF1α-stabilizing effect of HOIP. The effect of HOIP was markedly abolished when HOIL-1L and Sharpin were deleted (Fig. 1M). Notably, overexpression or knockdown of HOIL-1L or Sharpin alone had no significant impact on HIF1α expression (Fig. 1N, O and S1H-K). We then analyzed the effect of HOIP^C885A^, an E3 ligase-dead HOIP mutant [6], on HIF1α expression. This mutant had minimal, if any, impact on HIF1α expression (Fig. 1P). Finally, HOIPIN-8, a well-known inhibitor of LUBAC [45], efficiently blocked LUBAC-induced HIF1α up-regulation in A549^HOIPOE^ cells (Fig. S1L). Taken together, LUBAC stabilization of HIF1α requires its enzymatic activity.

### LUBAC potentiates HIF1α activity in a catalytic-dependent manner

**Fig. 2:**
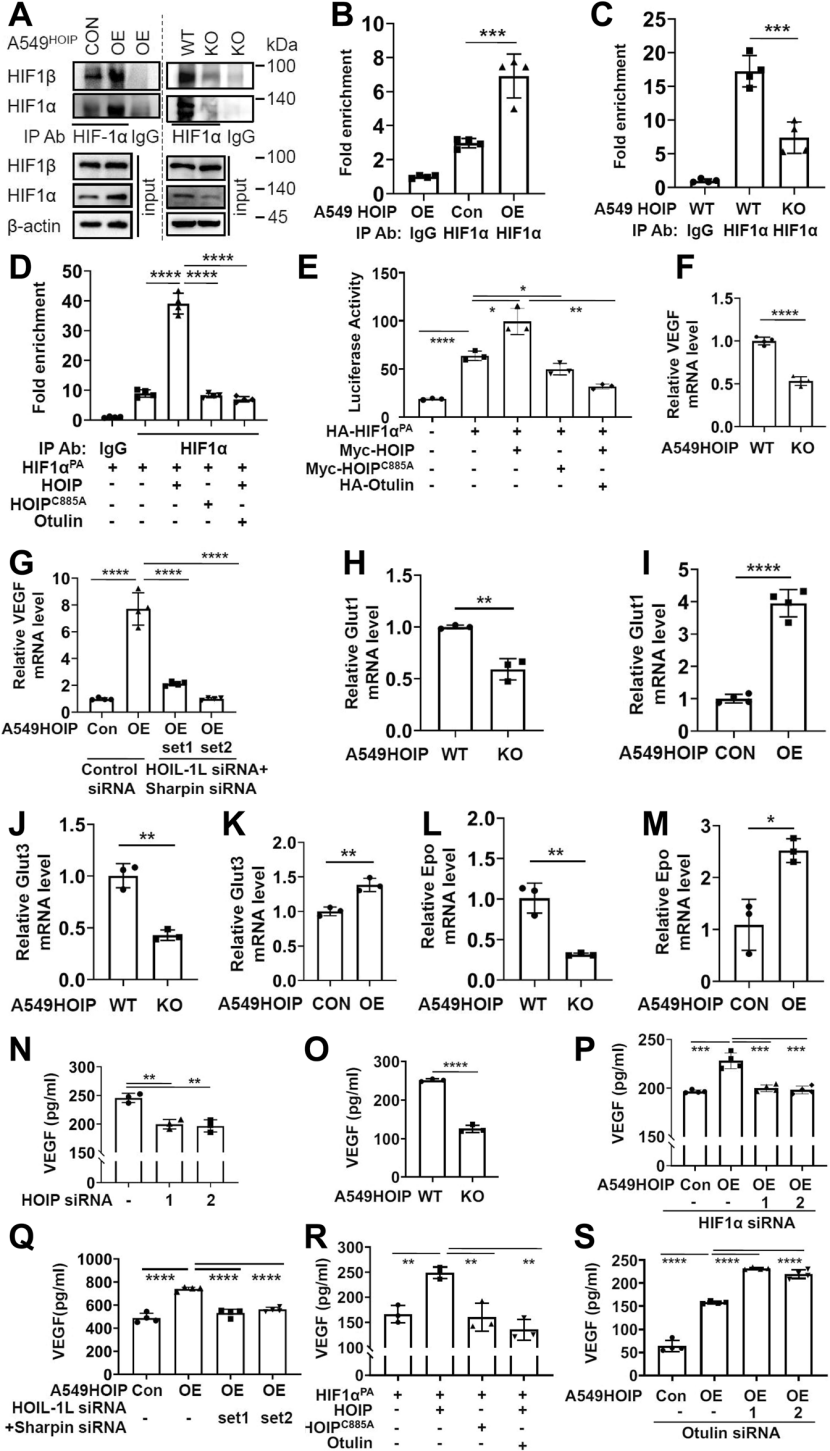
LUBAC-induced HIF1α activity requires its catalytic activity. **A** Immunoprecipitation/immunoblotting analysis of HIF1α-HIF1β dimerization in A549^HOIPCON^ and A549^HOIPOE^ (left panel) as well as A549^HOIPWT^ and A549^HOIPKO^ (right panel) cells. **B**–**D** ChIP analysis of HIF1α binding to the VEGF promoter in A549^HOIPCON^ and A549^HOIPOE^ (**B**), A549^HOIPWT^ and A549^HOIPKO^ (**C**), and HEK293T cells transfected as indicated (**D**). **E** Luciferase analysis of HIF1α transcriptional activity in HEK293T cells transfected as indicated. **F**, **G** qPCR analysis of the *VEGF* mRNA expression in A549^HOIPWT^ and A549^HOIPKO^ cells (**F**), as well as A549^HOIPCON^ and A549^HOIPOE^ cells transfected as indicated (**G**). **H**, **J**, **L** qPCR analysis of levels of the *Glut1* (**H**), *Glut3* (**J**) and *Epo* (**L**) mRNAs in A549^HOIPWT^ and A549^HOIPKO^ cells. **I**, **K**, **M** qPCR analysis of levels of the *Glut1* (**I**), *Glut3* (**K**) and *Epo* (**M**) mRNAs in A549^HOIPCON^ and A549^HOIPOE^ cells. **N**, **O** Measurement of VEGF production by A549 (**N**), as well as A549^HOIPWT^ and A549^HOIPKO^ (**O**) cells using ELISA. **P**, **Q** Measurement of VEGF production by A549^HOIPCON^ and A549^HOIPOE^ cells transfected with *HIF1α* siRNA (**P**) or *HOIL-1L* plus *Sharpin* siRNAs (**Q**) by ELISA. **R** Measurement of VEGF production by HEK293T cells transfected with as indicated by ELISA. **S** Measurement of VEGF production in A549^HOIPCON^ and A549^HOIPOE^ cells transfected as indicated by ELISA. Data are expressed as the mean ± SEM. *p < 0.05, **p < 0.01, ***p < 0.001, ****p < 0.0001 by paired 2-tailed Student’s *t*-test.

HIF1α activity is primarily regulated through modulating its expression [13, 14]. Having shown that LUBAC promotes HIF1α expression, we assessed the effect of LUBAC on its activities. We first showed that HOIP overexpression increased HIF1α-HIF1β interaction, while HOIP elimination decreased their dimerization in A549 cells (Fig. 2A). ChIP assays indicated HOIP overexpression and deletion in A549 cells increased and reduced HIF1α recruitment to the VEGF promoter (Fig. 2B, C), respectively. Importantly, overexpression of HOIP (but not its catalytically inactive mutant) heightened HIF1α binding to the promoter, which was substantially suppressed by Otulin (Fig. 2D and S2A). Luciferase assays showed that HOIP, rather than its catalytically inactive mutant, potentiated HIF1α transcription activity, which was markedly attenuated by Otulin (Fig. 2E and S2B). Therefore, LUBAC augments DNA-binding and transcriptional activities of HIF1α in a catalytic-dependent manner.

To further substantiate the impact of LUBAC on HIF1 transcriptional ability, we conducted qPCR to measure the effect of LUBAC on the expression of several well-known HIF1 targets encoding VEGF, glucose transporter 1 (Glut1), Glut3, and erythropoietin (Epo). *VEGF* mRNA was declined in A549^HOIPKO^ cells (Fig. 2F), but increased in A549^HOIPOE^ cells (Fig. 2G and S2D). Elimination of HOIL-1L and Sharpin in A549^HOIPOE^ cells abolished the ability of HOIP to up-regulate *VEGF* mRNA expression (Fig. 2G). HOIP further strengthened the effect of HIF1α to induce *VEGF* mRNA expression (Fig. S2C). Similarly, the expression of *Glut1, Glut3* and *Epo* mRNAs was decreased in A549^HOIPKO^ cells (Fig. 2H, J, L) but increased in A549^HOIPOE^ cells (Fig. 2I, K, M) when compared with their corresponding counterparts. Thus, LUBAC potentiates HIF1 transcriptional ability. Based on the dramatic effect of LUBAC on VEGF and our primary goal, this study was focused on the effect of LUBAC on VEGF.

We then employed ELISA to further examine the effect of LUBAC on VEGF production. HOIP silencing reduced VEGF production in A549 cells in hypoxia (Fig. 2N, O). A similar observation was achieved in NCI-H460 cells (Fig. S2E). In contrast, overexpression of HOIP in A549 cells promoted VEGF production (Fig. 2P; Fig. S2F), which was reverted upon HIF1α deletion (Fig. 2P). Also, elimination of HOIL-1L and Sharpin in A549^HOIPOE^ cells abrogated the stimulatory effect of HOIP on VEGF production (Fig. 2Q). Ectopic expression of HOIP, but not its inactive mutant, increased VEGF production; such induction was compromised by Otulin (Fig. 2R). In contrast, deletion of Otulin enhanced VEGF production in A549^HOIPOE^ cells (Fig. 2S) and NCI-H460 cells (Fig. S2G). Collectively, LUBAC precipitates VEGF expression and production through HIF1α, which is catalytic-dependent.

### LUBAC facilitates angiogenesis

**Fig. 3:**
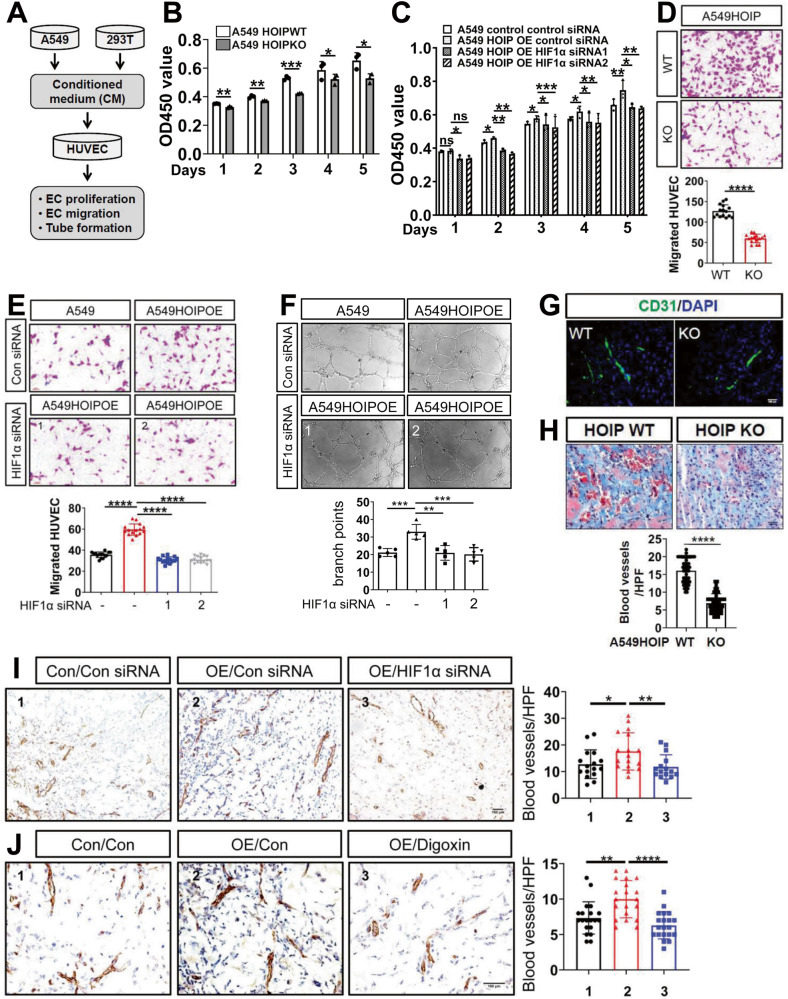
LUBAC facilitates angiogenesis. **A** Diagramed is the working model of the experiments depicted in this figure. **B**, **C** Proliferation of endothelial cells (EC) exposed to conditioned medium (CM) from A549^HOIPWT^ and A549^HOIPKO^ cells (**B**), as well as A549^HOIPCON^ and A549^HOIPOE^ cells transfected as indicated (**C**). **D**, **E** Migration of EC exposed to the CM from A549^HOIPWT^ and A549^HOIPKO^ cells (**D**), as well as A549^HOIPCON^ and A549^HOIPOE^ cells transfected as indicated (**E**). **F** Tube formation of EC exposed to the CM from A549^HOIPCON^ and A549^HOIPOE^ cells transfected with *HIF1α* (or control) siRNA. **G**, **H** Matrigel plugs of A549^HOIPWT^ and A549^HOIPKO^ cells were subjected to CD31 immunostaining (**G**), Masson’s trichrome staining (**H**) to evaluate micro-vessel density (**H**). **I**, **J** CD31 immunostaining and quantitation of the matrigel plugs of A549^HOIPCON^ and A549^HOIPOE^ cells treated with *HIF1α* (or control) siRNAs (**I**) or digoxin (or control) (**J**). Original magnification: 400 (**G**, **H**, **J**), 200 (**I**), and 100 (**D**–**F**). Scale bars: 100 μm (**D**–**H**), and 150 μm (**I**, **J**). Data are expressed as the mean ± SEM. *p < 0.05, **p < 0.01, ***p < 0.001, ****p < 0.0001 by paired 2-tailed Student’s *t*-test.

The most potent proangiogenic molecule VEGF can potently promote angiogenesis primarily through manipulating proliferation and migration of endothelial cells (EC) [13]. We collected VEGF-containing supernatants from cells overexpressing or lacking HOIP to obtain conditioned medium (CM) (Fig. 3A). We first evaluated the role for LUBAC in EC proliferation. A549^HOIPKO^–derived CM suppressed (Fig. 3B) but A549^HOIPOE^ CM promoted (Fig. 3C and S2H) EC proliferation. Deletion of HIF1α reversed HOIP effect (Fig. 3C). We then examined the role for LUBAC in EC migration. A549^HOIPKO^ CM retarded EC migration (Fig. 3D). In contrast, EC migration was precipitated in response to A549^HOIPOE^ CM (Fig. 3E and S2I), which was markedly impaired by HIF1α silencing (Fig. 3E). Thus, LUBAC promotes EC proliferation and migration in a HIF1α-dependent manner. As expected, endothelial tube formation was potently enhanced by A549^HOIPOE^ CM (Fig. 3F). Compared with A549^HOIPOE^ CM, the CM from A549^HOIPOE^ transfected with *HIF1α* siRNAs showed marginal effect on tube formation (Fig. 3F). In contrast, tube formation was declined by A549^HOIPKO^ CM (data not shown).

To further determine the role for LUBAC in angiogenesis, we conducted matrigel plug assays. Deletion of HOIP in A549 cells reduced angiogenesis in the plugs (Fig. 3G, H). HOIP overexpression stimulated plug angiogenesis (Fig. 3I, J), which was significantly compromised when cells treated with HIF1α siRNAs (Fig. 3I) and digoxin, a HIF1α inhibitor [46] (Fig. 3J). Collectively, LUBAC accelerates angiogenesis via HIF1α in a catalytic dependent manner.

### HIF1α selectively interacts with HOIP

**Fig. 4:**
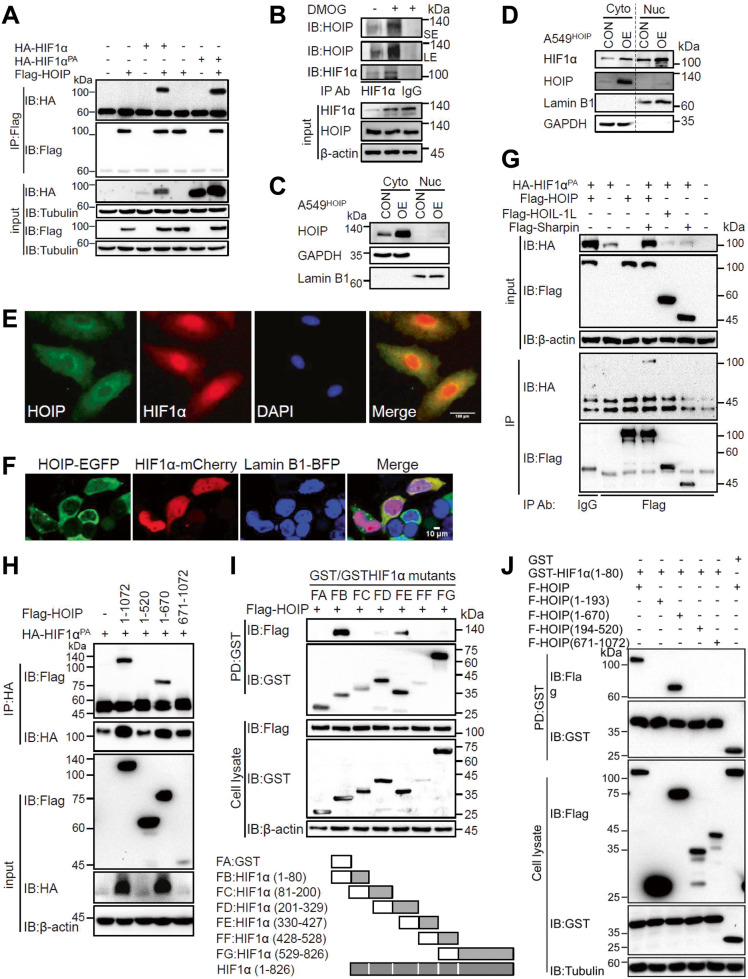
LUBAC physically interacts with HIF1α. **A** Co-immuoprecipitation (IP)/immunoblotting (IB) analysis of the interaction between HOIP and HIF1α or HIF1α^PA^ in HEK293T cells transfected as indicated. **B** IP/IB analysis of the interaction between endogenous HOIP and HIF1α in A549 treated as indicated. **C**, **D** Subcellular fractionation analysis of HOIP (**C**, **D**) and HIF1α (**D**) in A549^HOIPCON^ and A549^HOIPOE^ cells. The expression of GAPDH and Lamin B1 was included to verify the identity of the cytoplasmic (Cyto) and a nuclear (Nuc) proteins, respectively. **E** Analysis of co-localization of HOIP and HIF1α in A549 cells by immunofluorescence staining. **F** Confocal microscopy of HOIP-EGFP and HIF1α-mCherry in HEK293 cells transfected as indicated in Materials and Methods. **G** Co-IP/IB analysis of the interaction between HIF1α and LUBAC components in HEK293T cells transfected as indicated. **H** Co-IP/IB analysis of the interaction of HIF1α and full-length or different regions of HOIP in HEK293T cells transfected as indicated. **I**, **J** GST pulldown analysis of the interaction between HOIP and different fragments of HIF1α (**I**) or HIF1α and different fragments of HOIP (**J**) in HEK293T cells transfected as indicated.

**Fig. 5:**
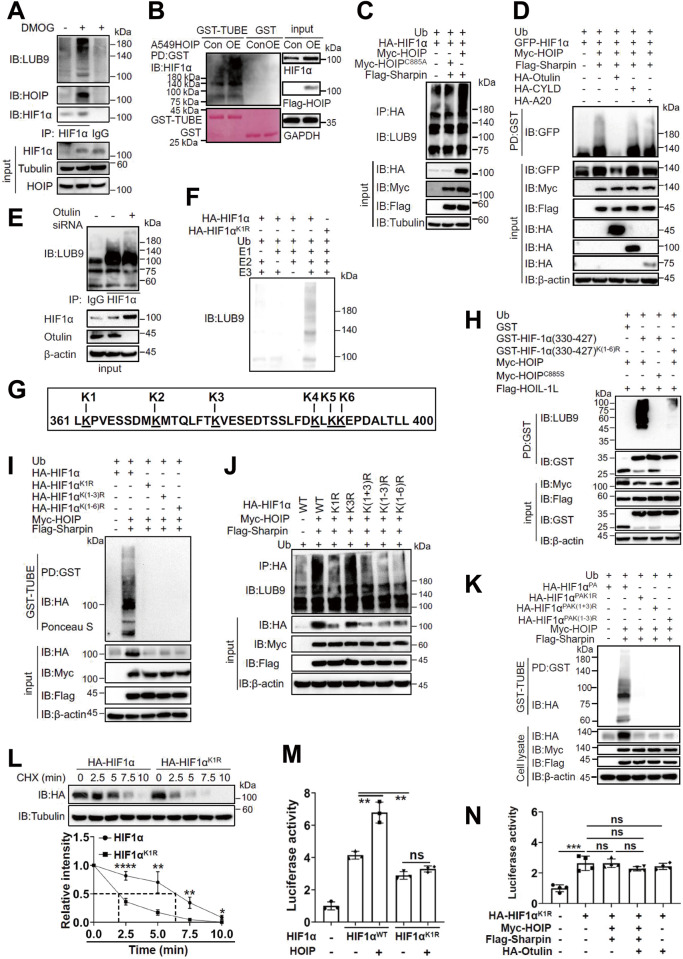
LUBAC catalyzes the linear ubiquitination of HIF1α at lysine 362. **A** Immuoprecipitation (IP)/immunoblotting (IB) analysis of the linear ubiquitination of endogenous HIF1α in A549 cells. **B** GST-TUBE analysis of HIF1α linear ubiquitination in A549^HOIPCON^ and A549^HOIPOE^ cells. **C** HIF1α linear ubiquitination in HEK293T cells transfected as indicated. **D** GST-TUBE analysis of HIF1α linear ubiquitination in transfected 293T cells in hypoxia. **E** Co-IP/IB analysis of the effect of *Otulin* siRNA on HIF1α linear ubiquitination in A549 cells. **F** In vitro linear ubiquitination of HIF1α. **G** Schematic shown of six lysine residues within HIF1α(330-427). **H** GST pulldown analysis of the linear ubiquitination of HIF1α(330-427) and its lysine-free mutant in HEK293T cells transfected as indicated. **I**, **J** GST-TUBE (**I**) and Co-IP/IB (**J**) analysis of the linear ubiquitination of HIF1α and its mutants in HEK293T cells transfected as indicated. **K** GST-TUBE analysis of the linear ubiquitination of HIF1α^PA^ and its mutants in HEK293T cells transfected as indicated. **L** Cycloheximide chase experiment monitoring HIF1α^K1R^ stability in HEK293T cells. **M**, **N** Luciferase assay for HIF1α^K1R^ transcriptional activity and its regulation by different proteins in HEK293T cells transfected as indicated. Data are expressed as the mean ± SEM. *p < 0.05, **p < 0.01, ***p < 0.001, ****p < 0.0001 by paired 2-tailed Student’s *t*-test.

Having documented that the catalytic activity of LUBAC is necessary for its effect on HIF1α stability and activity, we assumed that HIF1α is a novel substrate for LUBAC. We first examined whether LUBAC interacts with HIF1α. Co-immunoprecipitation (IP)/IB experiment showed that HOIP interacted with HIF1α (Fig. 4A). Similar finding was observed with HIF1α^PA^ (Fig. 4A). Together, unlike pVHL, LUBAC recognition of HIF1α does not require its prolyl hydroxylation. In A549 cells, endogenous HOIP and HIF1α interacted with each other, which was further enhanced by DMOG (Figs. 4B and 5A).

We next examined whether and where HOIP co-localizes with HIF1α in cells. It is well known that HIF1α is distributed in the cytoplasm and nucleus. We first performed subcellular fractionation assay to determine whether HOIP is cytosolic and/or nuclear. We found that HOIP is cytosolic (Fig. 4C). As anticipated, HIF1α was detected in both the cytoplasm and nucleus (Fig.4D). Cell fractionation assay implied that HOIP and HIF1α interacted with each other in the cytoplasm (Fig. 4D). We then conducted immunofluorescence staining to ascertain their co-localization. Indeed, we found that HOIP co-localized with HIF1α dominantly in the cytoplasm (Fig. 4E). Additionally, we pursued confocal microscopy to further affirm the above observations. We cotransfected vectors expressing HOIP-EGFP and HIF1α-mCherry into cells and showed that both fusion proteins were co-localized in the cytoplasm (Fig. 4F). Taken together, HOIP co-localizes with HIF1α in the cytoplasm.

Notably, HIF1α interacted with HOIP, but not HOIL-1L or Sharpin (Fig. 4G). The same observation was made in the reciprocal co-IP experiment (Fig. S3A). We next mapped the HOIP region responsible for HIF1α binding. HOIP(521-570) is instrumental for HIF1α binding (Fig. 4H). The same finding was achieved in the reciprocal co-IP experiment (Fig. S3B). We also determined HOIP-binding region within HIF1α. HOIP interacted with 3 different regions of HIF1α with aa1-80 being dominant domain (Fig. 4I and S3C, D). Interestingly, HIF1α(1-80) bound HOIP rather than HOIL-1L and Sharpin (Fig. S3C). As expected, a robust interaction occurred between HIF1α(1-80) and HOIP(521-570) (Fig. 4J). Together, HIF1α binds HOIP rather than HOIL-1L and Sharpin.

### LUBAC catalyzes the linear ubiquitination of HIF1α at lysine 362

We next assessed whether LUBAC targets HIF1α for linear ubiquitination using two well-appreciated approaches: anti-linear Ub chain antibody (anti-LUB9) and TUBE [47, 48]. Indeed, anti-LUB9 recognized linear Ub chain conjugated onto NF-кB essential modulator (NEMO) (Fig. S4A), a well-known substrate for LUBAC [6]. HIF1α underwent linear ubiquitination in A549 cells (Fig. 5A). Linear ubiquitination could occur in hypoxia (data not shown). GST-TUBE (Fig. S4B) but not GST alone could pull down linearly ubiquitinated HIF1α in A549 cells (Fig. 5B). However, HIF1α linear ubiquitination was barely detectable in HOIP-deficient A549 cells (Fig. S4C, D). Notably, HIF1α linear ubiquitination also occurred in macrophages (Fig. S4E). LUBAC (but not its catalytically inactive mutant) induced the linear ubiquitination and stability of HIF1α in hypoxia (Fig. 5C and S4F, G), which was efficiently abolished by Otulin (Fig. 5D and S4H); however, the deubiquitinases CYLD and A20 failed to do so (Fig. 5D). As shown in Fig. 5E, elimination of Otulin enhanced HIF1α linear ubiquitination in A549 cells.

We further conducted in vitro linear ubiquitination assay and found that HIF1α was linearly ubiquitinated, while omitting any of three enzymes (E1, E2 and E3) failed to assemble linear Ub chains on HIF1α (Fig. 5F). This is the case for HA-HIF1α^PA^ (Fig. S4I). These findings highlight that LUBAC targets HIF1α for linear ubiquitination.

We next sought to identify the residue(s) responsible for HIF1α linear ubiquitination. To this end, we first determined the regions accounting for linear ubiquitination. Extensive studies revealed that HIF1α(330-427) is the crucial region contributing to linear ubiquitination (Fig. S5A-G). Mutation of all 6 Ks within this region (Fig. 5G) into arginine (R) abolished linear ubiquitination of HIF1α(330-427) (Fig. 5H and S5F, G). Furthermore, mutation of the first three Ks, but not last three ones, abrogated the linear ubiquitination of both HIF1α(330-427) (Fig. S5F) and full-length HIF1α (Fig. 5I). We then replaced the first 3 Ks individually with R and found that mutation of K1, rather than K2 or K3, abrogated linear ubiquitination (Fig. 5J and S5G, H). The same findings were achieved with HIF1α^PA^ (Fig. 5K and S5I, J). In vitro linear ubiquitination assay indicated that there was negligible, if any, linear Ub chains attached onto HA-HIF1α^K1R^ and HA-HIF1α^PAK1R^ mutants (Fig. 5F and S4I). Collectively, LUBAC induces the linear ubiquitination of HIF1α at K362. In addition, our findings emphasize that unlike pVHL-dictating HIF1α ubiquitination, prolyl hydroxylation is not a prerequisite for HIF1α linear ubiquitination by LUBAC.

### Linear ubiquitination enhances the stability and activity of HIF1α

Having identified K362 as the linear ubiquitination site of HIF1α, we assessed whether linear ubiquitination increases the stability and activity of HIF1α employing the linear ubiquitination-resistant mutant HIF1α^K362R^ (herein termed HIF1α^K1R^ for simplicity). To this end, we performed the CHX and luciferase assays with HIF1α^K1R^. CHX assay indicated that the half-life of HIF1α^K1R^ was markedly curtailed compared with that of wild-type HIF1α (Fig. 5L). The transcription activity of HIF1α^K1R^ was much weaker than that of HIF1α. Neither LUBAC (Fig. 5M, N) nor Otulin (Fig. 5N) affected HIF1α^K1R^ transcriptional activity. Together, linear ubiquitination by LUBAC enhances the stability and activity of HIF1α.

### Linear ubiquitination stabilizes HIF1α by antagonizing the CMA-lysosome pathway

**Fig. 6:**
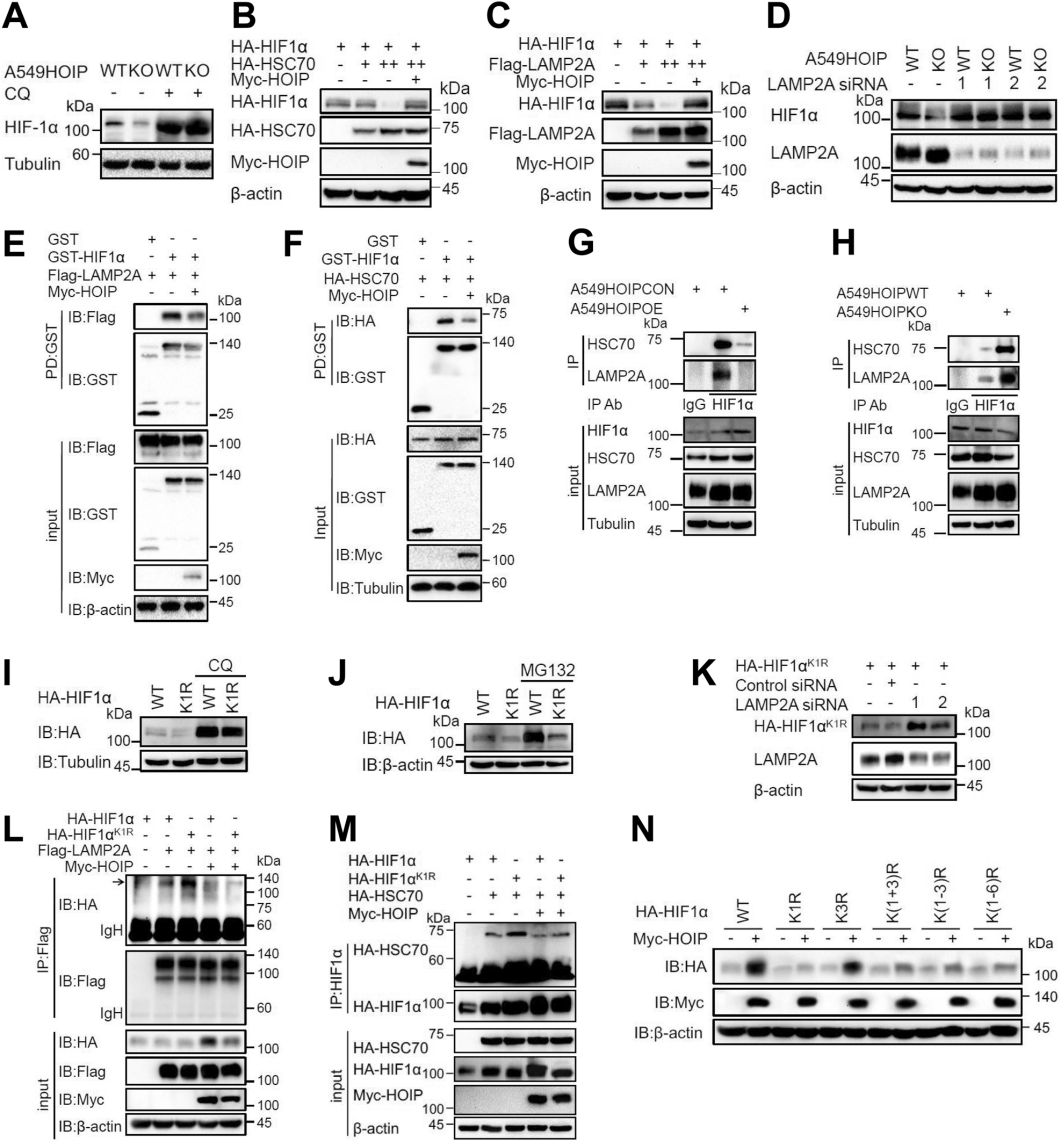
LUBAC stabilizes the HIF1α protein through antagonizing its degradation by the chaperone-mediated autophagy. **A** Immunoblotting (IB) analysis of the indicated proteins in A549^HOIPWT^ and A549^HOIPKO^ cells treated with chloroquine (CQ; 200 μM, 8 h) or PBS (control). **B**, **C** IB analysis of the indicated proteins in HEK293T cells transfected as indicated. **D** IB analysis of the indicated proteins in A549^HOIPWT^ and A549^HOIPKO^ cells transfected with control (-) or *LAMP2A* siRNAs. **E**, **F** GST pulldown analysis of the impact of HOIP on the HIF1α-LAMP2A (**E**) and HIF1α-HSC70 (**F**) interactions. **G**, **H** IP/IB analysis of the HIF1α-HSC70 and HIF1α-LAMP2A interactions in A549^HOIPCON^ and A549^HOIPOE^ (**G**) as well as A549^HOIPWT^ and A549^HOIPKO^ (**H**) cells. **I**, **J** HEK293T cells transfected with HIF1α and HIF1α^K1R^ were treated with chloroquine (CQ; 200 μM, 8 h) (**I**) or MG132 (20 μM, 8 h) (**J**), followed by IB analysis of the indicated proteins. **K** IB analysis of the indicated proteins in HEK293T cells transfected HIF1α with and HIF1α^K1R^ together with *LAMP2A* (or control) siRNAs. **L**, **M** Co-IP/IB analysis of HIF1α^K1R^ interaction with LAMP2A (**L**) and HSC70 (**M**) in HEK293T cells transfected as indicated. **N** IB analysis of the effect of LUBAC on the expression of HIF1α and its mutants indicated.

We attempted to dissect the mechanism whereby linear ubiquitination by LUBAC stabilizes HIF1α. HIF1α is degraded by proteasome and/or lysosome. Treatment of A549^HOIPKO^ cells with lysosome inhibitor chloroquine [32] (Fig. 6A), but not the proteasome inhibitor MG132 (Fig. S6A), restored HIF1α inhibition afforded by HOIP depletion. Moreover, LAMP2A siRNAs significantly increased HIF1α expression in A549 (Fig. S6B) and HEK293T (Fig. S6C) cells. LUBAC significantly reverted the HIF1α-degrading effects of HSC70 (Fig. 6B) and LAMP2A (Fig. 6C). In contrast, elimination of LAMP2A strongly rescued the inhibitory effect of HOIP depletion on HIF1α expression (Fig. 6D). HIF1α interacted with LAMP2A (Fig. S6D) and HSC70 (Fig. S6E). The HIF1α-LAMP2A and HIF1α-HSC70 interactions were significantly compromised by overexpressed HOIP in HEK293T cells (Fig. 6E, F) and in A549 cells (Fig. 6G). In stark contrast, deletion of HOIP increased the HIF1α-HSC70 and HIF1α-LAMP2A interactions in A549 cells (Fig. 6H). These data indicated that LUBAC stabilizes HIF1α by blunting the CMA-lysosome pathway.

We further delineated how LUBAC manipulates CMA-mediated HIF1α decay. We hypothesized that linear ubiquitination by LUBAC protects HIF1α against the destruction by the CMA-lysosome pathway. As expected, chloroquine (Fig. 6I) but not MG132 (Fig. 6J) substantially enhanced the expression of HIF1α^K1R^. Moreover, HIF1α^K1R^ expression was significantly increased by *LAMP2A* siRNAs (Fig. 6K). Intriguingly, HIF1α^K1R^ exhibited a much stronger interaction with LAMP2A (Fig. 6L) and HSC70 (Fig. 6M) than HIF1α, indicating that linear ubiquitination prevents HIF1α from interacting with HSC70 and LAMP2A. LUBAC had no significant effect on the expression of the HIF1α mutants with K1 being replaced by R including HIF1α^K1R^ (Fig. 6N). As a control, HIF1α^K3R^ expression was enhanced by HOIP as efficiently as that of HIF1α (Fig. 6N). Thus, K362 linear ubiquitination stabilizes HIF1α through antagonizing the CMA-lysosome pathway. Collectively, LUBAC retards HIF1α destruction via CMA through inducing HIF1α linear ubiquitination.

### LUBAC promotes proliferation, migration and invasion of lung cancer cells

**Fig. 7:**
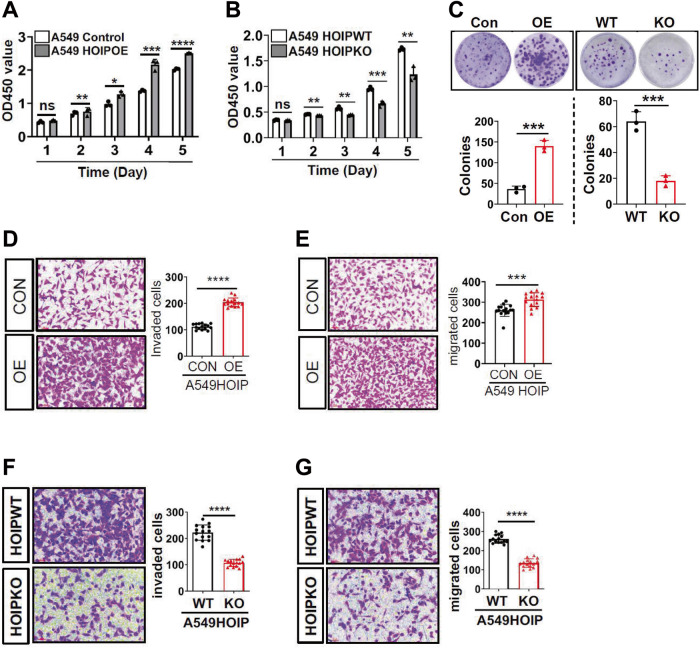
LUBAC promotes proliferation, clonogenic formation, migration and invasion of A549 lung cancer cells. **A**, **B** CCK8 assay for proliferation of A549^HOIPCON^ and A549^HOIPOE^ (**A**) as well as A549^HOIPWT^ and A549^HOIPKO^ (**B**) cells. **C** Clonogenic formation of A549^HOIPCON^ and A549^HOIPOE^ as well as A549^HOIPWT^ and A549^HOIPKO^ cells. **D,** **E** Transwell assay for invasion (**D**) and migration (**E**) of A549^HOIPCON^ and A549^HOIPOE^ cells. **F**, **G** Transwell assay for invasion (**F**) and migration (**G**) of A549^HOIPWT^ and A549^HOIPKO^ cells. Original magnification: 100 (**D**–**G**). Scale bars: 100 μm (**D**–**G**). Data are expressed as the mean ± SEM. *p < 0.05; **p < 0.01; ***p < 0.001; ****p < 0.0001 by paired 2-tailed Student’s *t*-test. NS, no significant difference.

HIF1 is also associated with distinct properties of cancers such as proliferation, invasion and migration. We found that A549^HOIPOE^ (Fig. 7A) and A549^HOIPKO^ (Fig. 7B) cells displayed an elevated and declined potential in proliferation, respectively. We then detected the capacity of LUBAC in regulating clonogenic formation of A549 cells. The ability of A549^HOIPOE^ cells to form colonies was increased, while A549^HOIPKO^ cells exhibited a decreased capacity of clonogenic formation (Fig. 7C). We also showed that overexpression of HOIP greatly facilitated invasion (Fig. 7D) and migration (Fig. 7E) of A549 cells. In sharp contrast, knockout of HOIP substantially reduced invasion (Fig. 7F) and migration (Fig. 7G) of A549 cells. Together, LUBAC increases proliferation, clonogenic ability, migration and invasion of lung cancer cells.

### LUBAC increases cancer burden in mice largely through regulating HIF1α

**Fig. 8:**
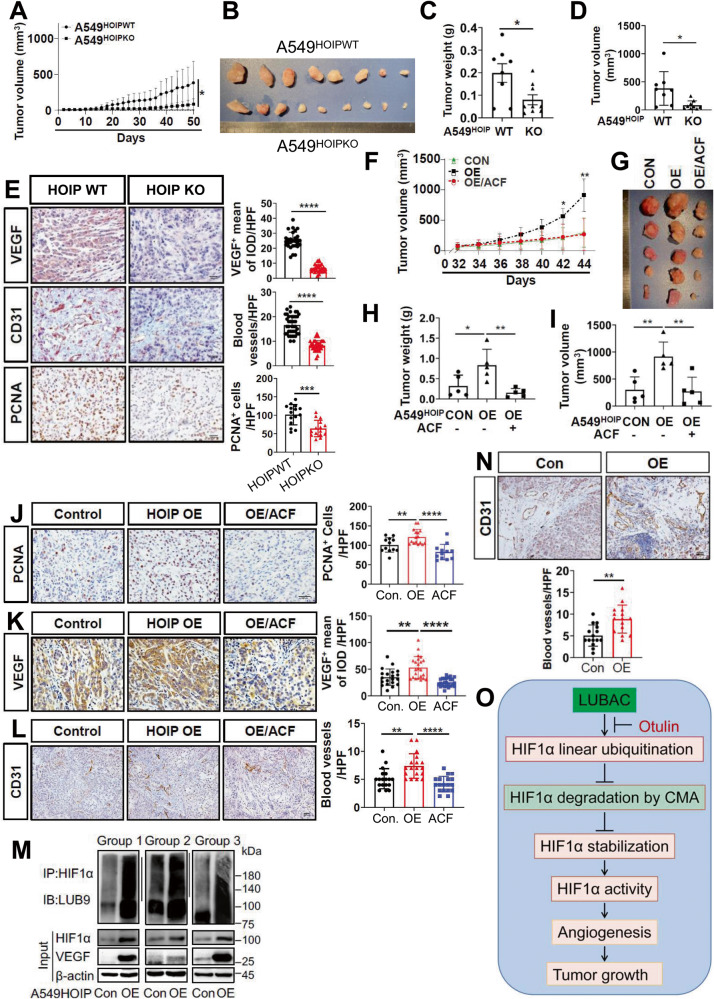
LUBAC exacerbates angiogenesis and growth of lung carcinoma in vivo. **A**–**E** A549^HOIPWT^ (*n* = 8) and A549^HOIPKO^ (*n* = 9) lung cancer cells were subcutaneously inoculated into nude mice. The mice were dissected 7 weeks after implantation. Shown are tumor growth rate (**A**), representative photograph of tumors (**B**), tumor weight (**C**) and volume (**D**); Immunohistochemistry analysis and quantification of VEGF expression (**E**), micro-vessel density (**E**), and the number of PCNA^+^ cells (**E**) in tumors. **F**–**L** A549^HOIPCON^ (*n* = 5) and A549^HOIPOE^ (*n* = 10) lung cancer cells were subcutaneously inoculated into nude mice. Two weeks after inoculation, mice bearing A549^HOIPOE^ tumors were randomly divided into two groups. Mice were daily administrated H_2_O (for A549^HOIPCON^ and A549^HOIPOE^; *n* = 5/group) or acriflavine (ACF) (for A549^HOIPOE^; 2 mg/kg body weight in H_2_O; *n* = 5) intraperitoneally for 2 weeks. Shown are tumor growth rate (**F**), representative photograph of tumors (**G**), and tumor weight (**H**), tumor volume (**I**), and immunohistochemistry analysis and quantification of the number of PCNA^+^ cells (**J**), VEGF expression (**K**), micro-vessel density (**L**). **M**, **N** Immunoblotting analysis of linearly ubiquitinated HIF1α and the indicated proteins in tumor tissues (*n* = 3) from nude mice inoculated A549^HOIPCON^ and A549^HOIPOE^ lung cancer cells for 10 weeks (**M**). The tumor tissues were examined for micro-vessel density (**N**). **O** Working model for LUBAC regulation of tumor angiogenesis and growth. Original magnification: 400 (**E**, **J**, **K**), and 200 (**L**, **N**). Scale bars: 100 μm (**E**), and 150 μm (**J**–**L**, **N**). Data are expressed as the mean ± SEM. *p < 0.05; **p < 0.01; ***p < 0.001; ****p < 0.0001 by paired or unpaired 2-tailed Student’s *t-*test.

To further verify and extend aforementioned findings, we determined the importance of HOIP in lung cancer in mouse xenograft model. We first inoculated A549^HOIPKO^ and A549^HOIPWT^ cells into nude mice and analyzed tumor tissues. Compared with those received A549^HOIPWT^ cells, mice carrying A549^HOIPKO^ cells had a much smaller tumor burden (Fig. 8A-D). The content of PCNA, a hallmark of cell proliferation, was much lower in tumors from mice carrying A549^HOIPKO^ cells than those from A549^HOIPWT^-inoculated mice (Fig. 8E). Tumors derived from A549^HOIPKO^ cells displayed a marked reduction in VEGF expression as well as micro-vessel density (Fig. 8E).

We next examined the influence of HOIP overexpression on angiogenesis and growth of lung cancer. Mice received A549^HOIPOE^ cells exhibited a dramatic increase in tumor burden compared with those bearing A549^HOIPCON^ cells (Fig. 8F–I). Remarkably, administration of acriflavine (ACF), a HIF1α inhibitor [49], to mice bearing A549^HOIPOE^ cells significantly mitigated tumor burden (Fig. 8F–I). Mice harboring A549^HOIPOE^ cells displayed a substantial increase in PCNA content (Fig. 8J), VEGF expression (Fig. 8K) and micro-vessel density (Fig. 8L) in comparison to those carrying A549^HOIPCON^ cells. These changes were greatly suppressed by ACF (Fig. 8J–L). Therefore, LUBAC exacerbates angiogenesis and growth of lung cancer, which are contingent largely on HIF1α.

We conducted another set of study to evaluate the impact of LUBAC on HIF1α linear ubiquitination, expression and activity in mice. Strikingly, HIF1α linear ubiquitination was evident in tumor tissues from nude mice inoculated A549^HOIPCON^ cells and further enhanced in A549^HOIPOE^ tumors (Fig. 8M), strengthening our findings achieved with cultured cells and cell free system presented earlier. Elevated HOIP expression robustly increased the expression of HIF1α and VEGF (Fig. 8M). In line with these results, micro-vessel density was significantly increased in tumor tissues with HOIP overexpression (Fig. 8N). Thus, LUBAC plays a causal role in promoting HIF1α expression and angiogenesis through catalyzing HIF1α linear ubiquitination in lung cancer.

## Discussion

Currently, we are far from a comprehensive understanding of LUBAC in cellular signaling [2]. The most important finding of this study is that LUBAC increases HIF1α activity and angiogenesis through inducing linear ubiquitination and stabilization of HIF1α, potentiating lung tumorigenesis (Fig. 8O). Therefore, this study provides novel insights into the role for LUBAC in HIF1 signaling and thus broadens our knowledge on LUBAC in cell signaling.

Our study reveals a role of linear ubiquitination in stabilization of HIF1α. Numerous studies on LUBAC suggested that linear ubiquitination plays instrumental roles in regulating cell signaling (particularly the NF-κB pathway) rather than protein stability [50]. A recent study reported that the assembly of linear Ub chain by LUBAC on misfolded Huntingtin promotes its proteasomal degradation [51]. LUBAC also induces the linear ubiquitination and stability of β-catenin [52], ATG13 [53] and selenoprotein GPX4 [54]. Together, our work and others indicate that linear ubiquitination by LUBAC is indispensable for protein quality-control.

HIF1α is a labile protein. O_2_-dependent hydroxylation of HIF1α accounts for the predominant mechanism whereby the alterations in O_2_ availability are transduced to HIF1-mediated changes in gene expression. In well-oxygenated cells, HIF1α is hydroxylated on P402 and P564 by the EGLN family of prolyl hydroxylases [17, 18]. Hydroxylation elicits HIF1α ubiquitination by pVHL and subsequent degradation in the proteasome [17, 18]. The EGLN hydroxylases absolutely require oxygen for their full enzymatic activity [18]. Under hypoxia, the EGLN hydroxylases become inactivated and HIF1α hydroxylation is abolished, resulting in HIF1α accumulation and nuclear translocation [18]. While the UPS is the best-appreciated mechanism controlling the stability of the HIF1α protein [18], emerging evidence demonstrated that CMA contributes significantly to HIF1α proteolysis in lysosome [20, 21]. Obviously, HIF1α protein turnover by the UPS and CMA occurs in the cytoplasm. In line with this notion, our study clearly demonstrated that HOIP selectively interacts with HIF1α primarily in the cytoplasm, inducing HIF1α linear ubiquitination. Linear ubiquitination shields HIF1α from CMA-mediated destruction in lysosome. Upon stabilization, HIF1α then translocates from the cytoplasm into the nucleus, where it dimerizes with HIF1β and binds to the HREs in the target genes to activate transcription [19–21].

Although the regulatory mechanisms underlying HIF1α decay by the UPS have been intensively studied, virtually nothing is known regarding the regulation of CMA in HIF1α homeostasis thus far. We showed that LUBAC-mediated linear ubiquitination confers protection against CMA-dependent destruction of HIF1α, thereby increasing HIF1α stability and activity. This study identifies LUBAC as the key regulator for CMA-dependent proteolysis of HIF1α. In addition, we provided solid evidence showing that LUBAC increases the linear ubiquitination and activities of wild-type HIF1α and its hydroxylation-resistant mutant to a similar extent. It is reasonable to conclude that unlike pVHL, LUBAC operates its functions independently of HIF1α prolyl hydroxylation. Thus, LUBAC provides an additional layer of regulation for HIF1α.

LUBAC is involved in embryonic vascularization [12, 29, 30]. A recent study demonstrated that fine-tuning linear ubiquitination of activin receptor-like kinase by LUBAC and Otulin controls embryonic vascularization [30]. Nonetheless, it is unknown regarding the role of LUBAC and linear ubiquitination in tumor angiogenesis to date. Our current study filled this gap by uncovering that LUBAC regulates angiogenesis through the HIF1-VEGF cascade. Given the instrumental role for HIF1-VEGF pathway in tumor angiogenesis [13, 14, 55], this study assigns a critical role for LUBAC in angiogenesis in lung cancer.

Our study indicated that LUBAC aggravates lung cancer growth in a HIF1-dependent manner. HIF1 has been linked to multiple facets of cancer properties, such as cancer stem cell specification, epithelial-mesenchymal transition, and metabolism reprogramming [19]. It will be intriguing to delve into the role and mechanism of LUBAC-mediated HIF1α linear ubiquitination in other processes in the future.

In conclusion, LUBAC is crucial regulator of HIF1α homeostasis that was not identified previously. Our work also sheds new light on the mechanism underlying LUBAC in regulating HIF1α homeostasis, tumor angiogenesis and lung tumorigenesis, making LUBAC an attractive therapeutic target for lung carcinoma.

### Supplementary information

Supplementary Figure Legends

LUBAC increases the protein but not mRNA levels of HIF1a.

The effect of LUBAC on HIF1a activities.

Interaction of HOIP with HIF1a.

LUBAC induces the linear ubiquitination of HIF1a.

Identification of the key residue responsible for HIF1a linear ubiquitination.

LUBAC stabilizes HIF1a protein through antagonizing its degradation via the chaperone-mediated autophagy.

## Data Availability

All data generated or analyzed during the current study are available from the corresponding authors upon reasonable request.
