# Supplementary material for: LUBAC promotes angiogenesis and lung tumorigenesis by ubiquitinating and antagonizing autophagic degradation of HIF1α
Source: Oncogenesis. 2024 Jan 25;13(1):6. doi: 10.1038/s41389-024-00508-3 (PMC10810860; doi:10.1038/s41389-024-00508-3)
Supplement: Supplementary file 1 — Supplementary Figure Legends [file 41389_2024_508_MOESM1_ESM.pdf]

Ying Jin, et al. Supplementary Materials

Supplementary Figure Legends

Figure S1. LUBAC increases the protein but not mRNA levels of HIF1α. A and **B** IB analysis of the expression of HIF1α (**A**) and HIF1α^{PA} (**B**) in HEK293T cells transfected as indicated. **C** Immunoblotting (IB) analysis of the indicated proteins in NCI-H460 cells transfected as indicated. **D** IB analysis of the indicated proteins in mouse macrophages. **E** IB analysis of the indicated proteins in NCI-H460 cells transfected as indicated. **F** qPCR analysis of the effect of *Otulin* siRNA on *HIF1α* mRNA expression in A549 cells. **G-I** IB analysis of the indicated proteins in HEK293T cells transfected as indicated. **J** and **K** IB analysis of the indicated proteins in A549 cells transfected with the indicated siRNAs. **L** IB analysis of the indicated proteins in A549^{HOIPCON} and A549^{HOIPOE} cells treated without (-) or with LUBAC inhibitor HOIPIN-8 (10 μM, 16 h).

Figure S2. The effect of LUBAC on HIF1α activities. **A** ChIP analysis of the ability of HIF1α to bind to the VEGF promoter in HEK293T cells transfected as indicated. **B** Luciferase assay for the effect of HOIP on HIF1α transcriptional activity in HEK293T cells transfected as indicated. **C** and **D** Real-time PCR analysis of *VEGF* mRNA levels in HEK293T cells transfected as indicated (**C**), as well as A549^{HOIPCON} and A549^{HOIPOE} cells (**D**). **E** and **G** ELISA analysis of VEGF production by NCI-H460 cells transfected with *HOIP* (or control) siRNA (**E**) or *Otulin* (or control) siRNA (**G**). **F** ELISA analysis of VEGF production by A549^{HOIPCON} and A549^{HOIPOE} cells. **H** and **I** Proliferation (**H**) and migration (**I**) of endothelial cells (EC) exposed to conditioned medium (CM) from A549^{HOIPCON} and A549^{HOIPOE} cells. Original magnification: 100 (**I**). Scale bars: 100 μm (**I**). Data are expressed as the mean±SEM. *$p < 0.05$, **$p < 0.01$, ***$p < 0.001$, ****$p < 0.0001$ by paired 2-tailed Student's *t* test.

Figure S3. Interaction of HOIP with HIF1α. A Co-immuoprecipitation (IP)/immunoblotting (IB) analysis of the interaction between HIF1α and LUBAC components in HEK293T cells transfected as indicated. **B** Co-IP/IB analysis of the interaction of HIF1α and full-length or different regions of HOIP in HEK293T cells transfected as indicated. **C** and **D** GST pulldown analysis of the interaction between HOIP and HIF1α (1-80) (**C**) or between HOIP and HIF1α(330-427) (**D**) in HEK293T cells transfected as indicated.

Figure S4. LUBAC induces the linear ubiquitination of HIF1α. **A** GST pulldown analysis of the specificity of anti-LUB9 antibody in HEK293T cells transfected as indicated. **B** Coommassie blue staining of purified GST-TUBE (0-5 μl purified protein). **C, D** Immunoprecipitation/immunoblotting (**C**) and GST-TUBE (**D**) analyses of HIF1α linear ubiquitination in A549^{HOIPWT} and A549^{HOIPKO} cells. **E** GST-TUBE analysis of the linear ubiquitination of HIF1α in J774A mouse macrophages. **F-H** GST-TUBE analysis of HIF1α^{PA} linear ubiquitination in HEK293T cells transfected as indicated. **I** In vitro linear ubiquitination of HIF1α^{PA} and its mutant.

Figure S5. Identification of the key residue responsible for HIF1α linear ubiquitination. **A-E** GST pulldown analysis of the linear ubiquitination of the different regions of HIF1α in HEK293T cells transfected as indicated. **F** and **G** GST pulldown analysis of the linear ubiquitination of HIF1α(330-427) and its lysine-free mutant in HEK293T cells transfected as indicated. **H** GST-TUBE analysis of the key residue responsible for the linear ubiquitination of HIF1α in HEK293T cells transfected as indicated. **I** and **J** Co-immunoprecipitation/immunoblotting analysis of the key residue responsible for the linear ubiquitination of HIF1α^{PA} in HEK293T cells transfected as indicated.

Figure S6. LUBAC stabilizes HIF1α protein through antagonizing its degradation via the chaperone-mediated autophagy. **A** Immunoblotting (IB) analysis of the indicated proteins in A549^{HOIPWT} and A549^{HOIPKO} cells treated with MG132 (10 μM) or DMSO for 8 h. **B** and **C** IB analysis of the indicated proteins in A549 (**B**) and HEK293T (**C**) cells transfected with control or *LAMP2A* siRNAs. **D** Co-immuoprecipitation (IP)/IB analysis of the interaction between HIF1α and LAMP2A in HEK293T cells transfected as indicated. **E** GST pulldown analysis of the HIF1α-HSC70 interaction in HEK293T cells transfected as indicated.
