# Supplementary figures and images for: LUBAC promotes angiogenesis and lung tumorigenesis by ubiquitinating and antagonizing autophagic degradation of HIF1α
Source: Oncogenesis. 2024 Jan 25;13(1):6. doi: 10.1038/s41389-024-00508-3 (PMC10810860; doi:10.1038/s41389-024-00508-3)

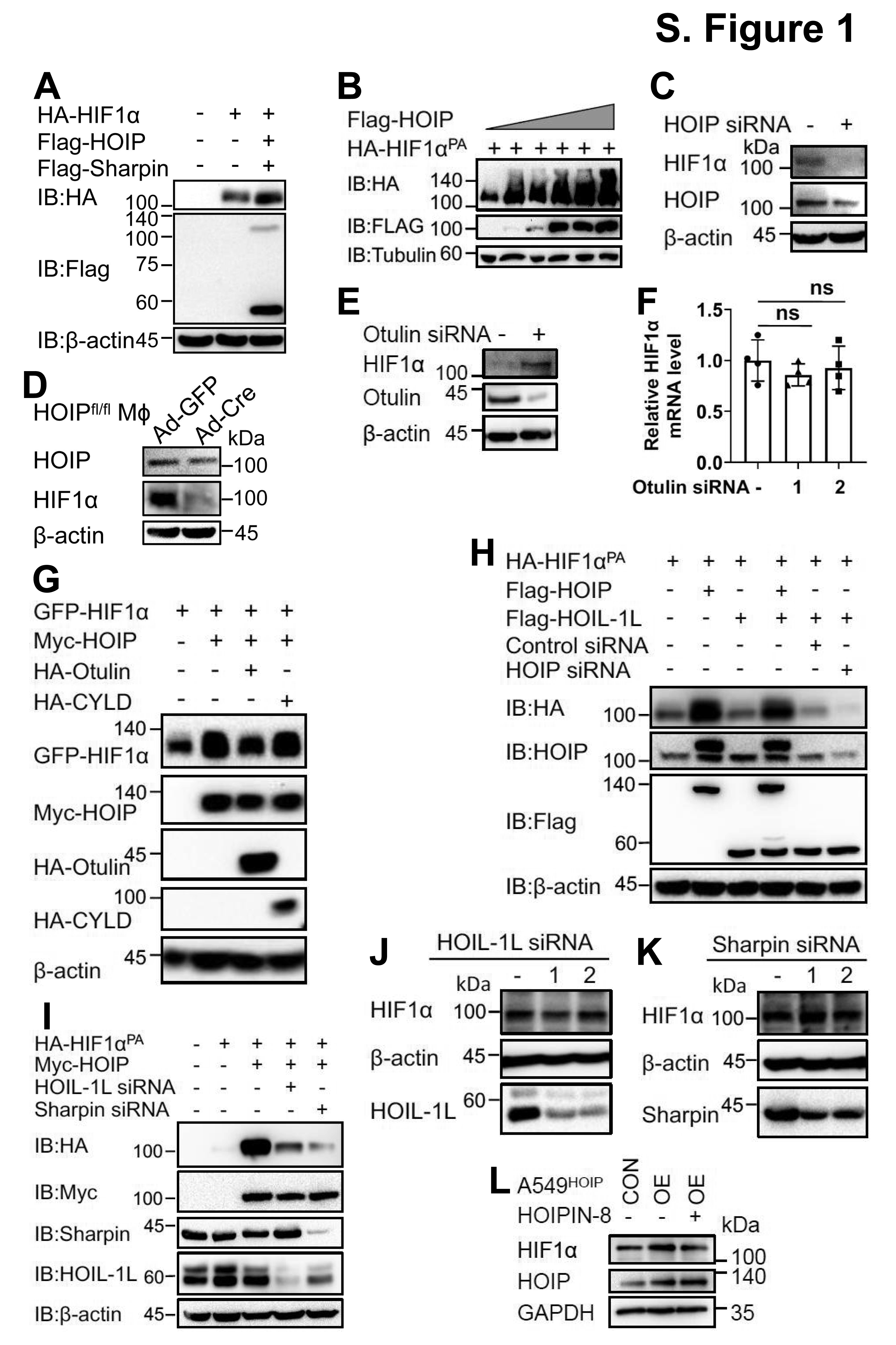

Supplement: Supplementary file 2 — LUBAC increases the protein but not mRNA levels of HIF1a. [file 41389_2024_508_MOESM2_ESM.tif]

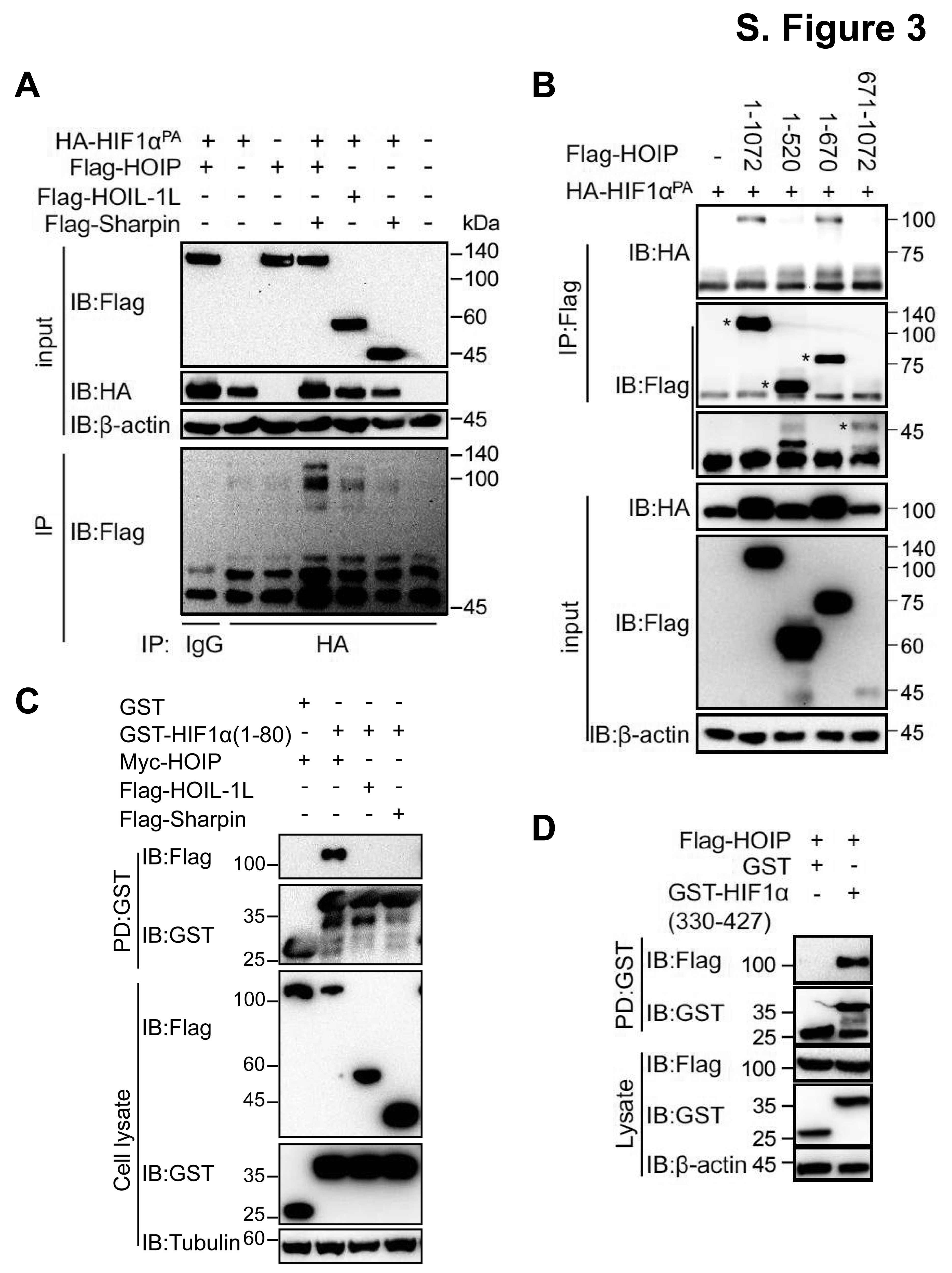

Supplement: Supplementary file 4 — Interaction of HOIP with HIF1a. [file 41389_2024_508_MOESM4_ESM.tif]

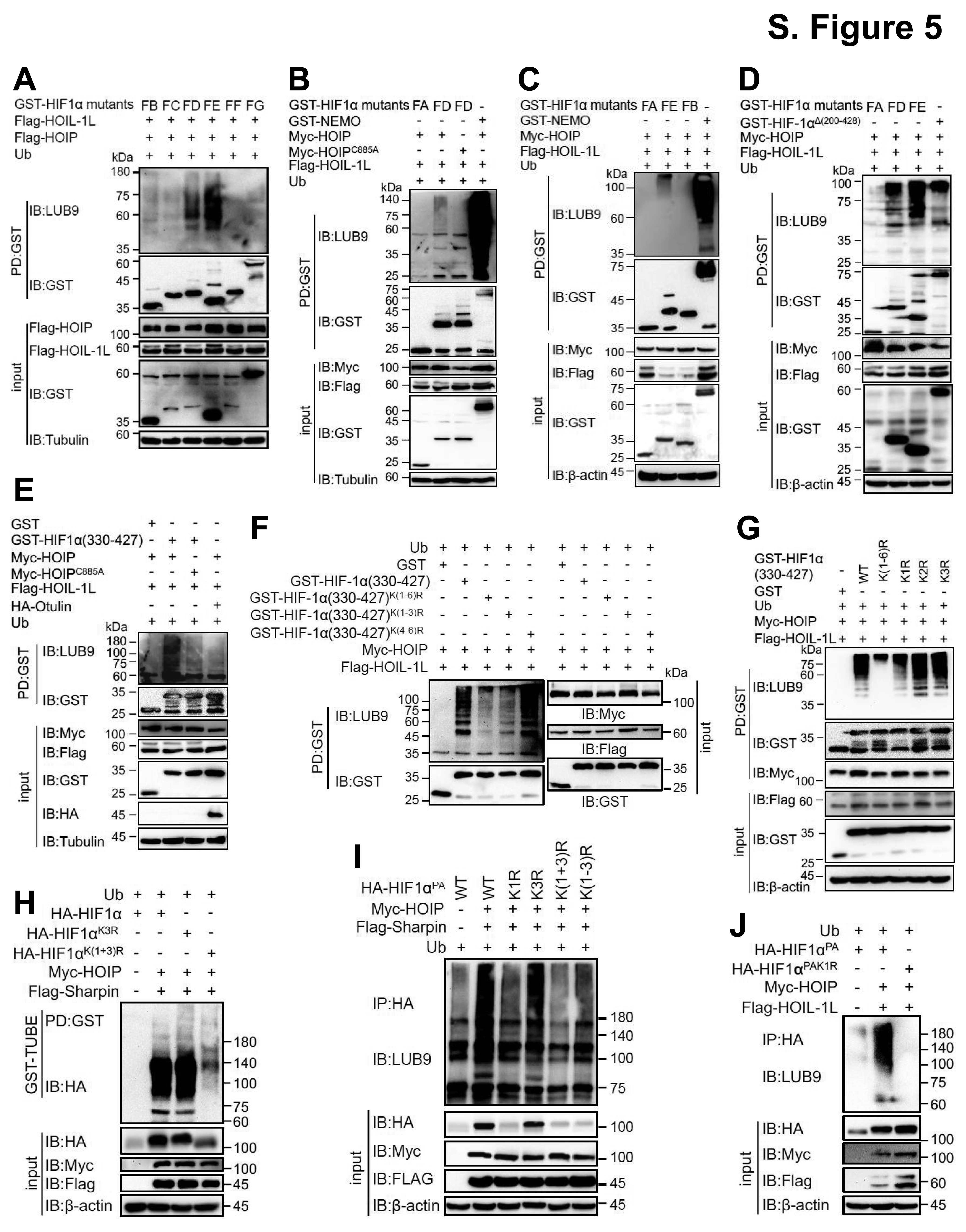

Supplement: Supplementary file 6 — Identification of the key residue responsible for HIF1a linear ubiquitination. [file 41389_2024_508_MOESM6_ESM.tif]

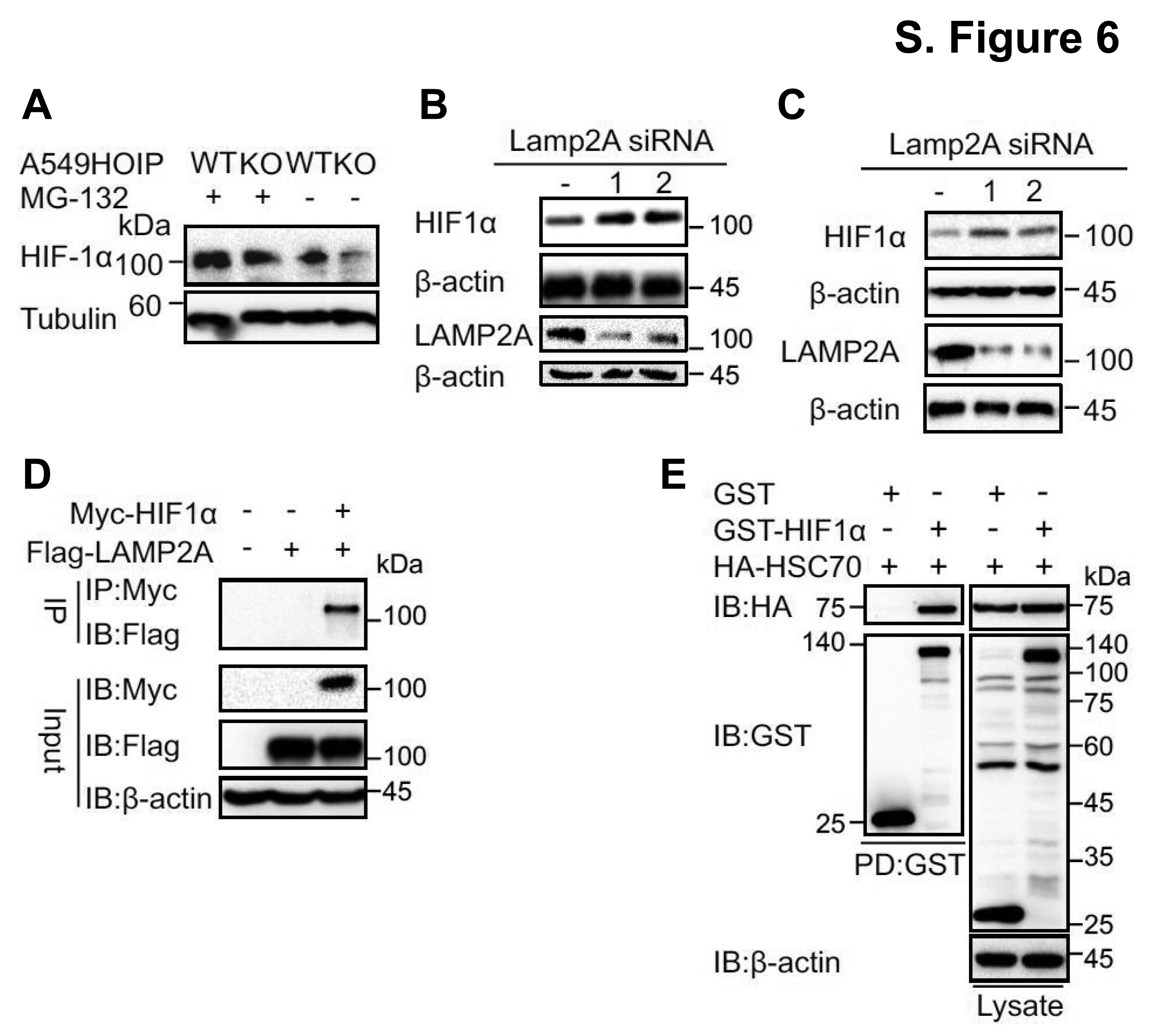

Supplement: Supplementary file 7 — LUBAC stabilizes HIF1a protein through antagonizing its degradation via the chaperone-mediated autophagy. [file 41389_2024_508_MOESM7_ESM.tif]
